# Metal Complexes Containing Natural and Artificial Radioactive Elements and Their Applications

**DOI:** 10.3390/molecules190810755

**Published:** 2014-07-24

**Authors:** Oxana V. Kharissova, Miguel A. Méndez-Rojas, Boris I. Kharisov, Ubaldo Ortiz Méndez, Perla Elizondo Martínez

**Affiliations:** 1 Facultad de Ciencias Químicas, Universidad Autónoma de Nuevo León, Ciudad Universitaria, Monterrey, N.L. C.P. 66450, Mexico; 2 Departamento de Ciencias Químico-Biológicas, Universidad de las Américas Puebla, Sta. Catarina Mártir, Cholula, Puebla. C.P. 72810, Mexico

**Keywords:** metal complex, coordination bond, organometallics, radionuclide, labeling, technetium, actinides

## Abstract

Recent advances (during the 2007–2014 period) in the coordination and organometallic chemistry of compounds containing natural and artificially prepared radionuclides (actinides and technetium), are reviewed. Radioactive isotopes of naturally stable elements are not included for discussion in this work. Actinide and technetium complexes with O-, N-, N,O, N,S-, P-containing ligands, as well π-organometallics are discussed from the view point of their synthesis, properties, and main applications. On the basis of their properties, several mono-, bi-, tri-, tetra- or polydentate ligands have been designed for specific recognition of some particular radionuclides, and can be used in the processes of nuclear waste remediation, *i.e.*, recycling of nuclear fuel and the separation of actinides and fission products from waste solutions or for analytical determination of actinides in solutions; actinide metal complexes are also usefulas catalysts forcoupling gaseous carbon monoxide, as well as antimicrobial and anti-fungi agents due to their biological activity. Radioactive labeling based on the short-lived metastable nuclide technetium-99m (^99m^Tc) for biomedical use as heart, lung, kidney, bone, brain, liver or cancer imaging agents is also discussed. Finally, the promising applications of technetium labeling of nanomaterials, with potential applications as drug transport and delivery vehicles, radiotherapeutic agents or radiotracers for monitoring metabolic pathways, are also described.

## Introduction

The actinide elements have unique physical and chemical properties related to their low-lying 7*p*, 6*d*, and 5*f* orbitals. Their more common oxidation state is +3, with general electronic configurations 7*p* 6*d* 5*f*^n^ (where n = 0, 1, 2…, 14). According to this definition, actinium, element 89, is the first member of the actinide series of elements, although it has no 5*f* electrons in its metallic, gaseous, or ionic forms [1]. Technetium (element 43) possesses two radioactive isotopes with long half-lives: ^99^Tc (2.12 × 10^5^ years, has practical use) and ^98^Tc (1.5 × 10^6^ years, it is a rhenium analogue). Both, actinides and ^99^Tc, although able to form metal complexes with ligands containing O-, N-, S- or P- heterodonor atoms, have different coordination chemistry, which is largely due to the differences in size and electronic structure. However, given their radioactive nature and their common uses as radiotracers, fuels, catalysts or radiopharmaceuticals, it makes sense to review the recent literature on their particular preparation methods and physical and chemical characteristics.

Actinides and technetium have numerous applications, but their uses strongly depend on the individual properties of each element. In particular, ^225^Ac, which has a 10-day half-life, is a potential agent for α-particle therapy, as it decays emitting three such particles [2]. On the contrary, curium is an underexplored element for a variety of reasons. ^244^Cm has a short half-life of 18 years and radiation damage in its compounds is very rapid. ^242^Cm was also available, but is even shorter-lived with a half-life of 163 days. Actinide-containing nanocrystals are currently also an object of intensive investigations [3,4,5,6].

Among the natural and artificial radioactive elements (Tc, Pm, Po, Fr, Ra, Ac and actinides), coordination and organometallic compounds of only technetium and the actinide series (An) are well represented at the present time [7,8,9,10]. The interest on their metal complexes has been motivated by the extended use of Tc, available in kilogram amounts, for medical and technical purposes. Meanwhile, actinides are important on their own for the nuclear industry and extraction and analytical applications [11,12,13]. They are also used for isotope separation, as antibacterial materials, drug delivery and anticancer radiopharmaceuticals, for the creation of labeled biomolecules, and radiation-induced processes. In the present review, dedicated to the coordination and organometallic chemistry of the actinides and Tc, we intended to present the synthetic techniques for these compounds according with the ligand nature.

## Part A. Actinides Chemistry

### A.1. General Concepts on Actinide Complexes

The electronic states of actinide atoms and ions are significantly different from those of lanthanides. In both series the successive filling of the *f* level proceeds up to the *f*^14^ configuration, but in the actinide series the filling only formally starts with Th, which has no *f* electrons and is the electronic analog of Hf. In contrast to lanthanides, the actinides display a wide collection of oxidation states. As An^3+^ ions they are analogs of the related Ln^3+^ ions, but as An^4+^ they resemble both Hf(IV) and Ce(IV) compounds. Actinides form various An^m+^ (m = 2–4) and AnO_2_^m+^ (m = 1, 2) ions containing only *f* electrons. The shielding by *f* electrons causes the contraction of the An^3+^ ions and the magnitude of the actinide contraction along the series to be parallel to that of the lanthanide contraction. Differences in correlation between the energy of 5*f* and 6*d*, as well as the 4*f* and 5*d*, levels lead to noticeable differences in the magnetic properties and electronic spectra of Ln^m+^ and An^m+^ ions. Technetium (4s^2^4p^6^4d^5^5s^2^ or 4s^2^4p^6^4d^6^5s^1^) has oxidation states from +1 to +7, however, those from +4 to +7 are the most stable.

Spin-orbit coupling (*J*) for the An^3+^ ions is very strong (2,000–4,000 cm^−1^) and larger that those for the Ln^3+^ ions (*ca.* 1,000 cm^−1^). In contrast to lanthanides, *J* is comparable with the ligand-field splitting and is no longer a good quantum number. The proximity of the energy of the 5*f* and 6*d* orbitals and the population of thermally accessible excited levels lead to the expression for effective magnetic moment μ_e_ = g[J(J + 1)]^1/2^ being appropriate for the lanthanides, but not for actinides.

Actinide organometallic complexes are compounds containing an actinide-carbon π-bond, an actinide-carbon σ-bond, or a combination of both. Actinide organometallic complexes are known for all of the early actinide elements (An) from thorium through californium. However, the majority of the reported data is on Th and U organometallic chemistry due to the extremely long half-lives of commercially available ^232^Th (in the form of ThCl_4_) and ^238^U (as UCl_4_) (1.41 × 10^10^ and 4.468 × 10^9^ years, respectively). Actinides have large metal and ionic radii and, therefore, large coordination numbers (*CN*) of up to 15. The uranium 6*d* orbitals play the primary role in covalent bonding between the metal and the ligand, while the 5*f* orbitals have a secondary role.

In contrast to lanthanides, the actinides have a variety of oxidation states in aqueous solution. The stable oxidation states go from +3 for Ac to +6 for U and Np and then successively decrease to +3 for Am and succeeding elements except No(+2). The maximum and stable oxidation states coincide for Ac, Th, Pa, U, Md, and Lr. Stable states are +7 for Np and Pu, +6 for Am, +4 for Cm, Bk, Cf, Es, and Fm, and +3 for No. The unstable (except for No and Md) oxidation state +2 is known for nearly all actinides in aqueous solution. The An^2+^, An^3+^, An^4+^, AnO^2+^, and AnO_2_^2+^ hydrated ions are known, which act as *Brönsted* acids.The An^4+^ cations are characteristic for actinides from Th through Cf (U^4+^ is readily oxidized) and in the case of Th is the only one existing in solution. Their acidity decreases in the order Pa^4+^ >> U^4+^ > Pu^4+^ > Np^4+^ > Th^4+^. The monoatomic ions exist only in very dilute solutions and tend to form polynuclear species when the concentration is increased. The acidity of the An^n+^ ions depends on the charge and radius of the central atom, so the An^4+^ and AnO_2_^2+^ ions are much stronger acids than An^3+^ and AnO_2_^+^, respectively. The redox behavior of the actinides is complicated by their high radioactivity, leading, in particular, to formation of H_2_O_2_ in aqueous solutions.

Moessbauer spectroscopy is a very useful tool to deduce the oxidation state and symmetry of the ligand environment. The gamma-resonance effect is observed for ^232^Th, ^231^Pa, ^238^U, ^24^^0^Pu, ^243^Am, and especially for ^237^Np with a ^237^U source. The isomer shifts for Np(VII) compounds are the largest (up to −70 mm/s) and decrease to +30 mm/s for Np(III).

### A.2. Actinide Complexes with O-Containing Ligands

**Figure 1:**
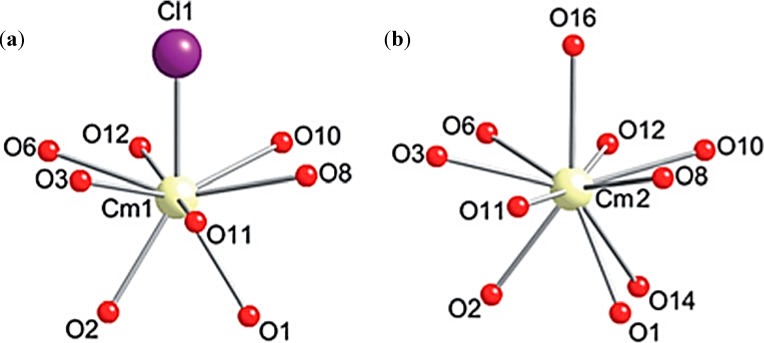
Views of two different coordination environments of Cm^III^ sites in Cm_2_[B_14_O_20_(OH)_7_(H_2_O)_2_Cl] with resolved disorder. (**a**) Nine-coordinated Cm(III) complex; (**b**) Ten-coordinated Cm(III) complex.

Although actinide metal complexes with the simplest inorganic ligands like water and anions were well studied in the previous century, sometimes novel and fresh ideas and calculation results on their structures and properties appear in the available literature. Thus, Car-Parrinello molecular dynamics simulations were used to examine the hydration structures, coordination energetics, and the first hydrolysis constants of Pu^3+^, Pu^4+^, PuO_2_^+^, and PuO_2_^2+^ ions were determined in aqueous solution at 300 K. It was found that the hexavalent PuO_2_^2+^ species are coordinated to five aquo ligands while the pentavalent PuO_2_^+^ complex is coordinated to four aquo ligands. The Pu^3+^ and Pu^4+^ ions are both coordinated to eight water molecules. The first hydrolysis constants obtained for Pu^3+^ and PuO_2_^2+^ are 6.65 and 5.70, respectively, all within 0.3 pH unit of the experimental values (6.90 and 5.50, respectively) [14]. Among other simple ligands, carbonates and borates have been also studied. Thus, curium(III) is able to form a stable complex in a high ionic strength aqueous solution, in the temperature range of 10–70 °C, as demonstrated recently by a time-resolved laser-induced fluorescence spectroscopy study [15]. Borate complex Cm_2_[B_14_O_20_(OH)_7_(H_2_O)_2_Cl] was synthesized [16] in autoclave using ^248^CmCl_3_ (3% ^246^Cm) and boric acid as the starting materials. Its crystallographic (Figure 1) and spectroscopic studies provided complementary information about this complex Cm^III^ borate. Both confirmed two distinct sites that are averaged in the crystal structure. It was hypothized that actinide borate compounds yield very distinct chemistry among 5*f* elements because of the large polarizability of the BO_3_ units. This yields unusual bonding with 5*f* orbitals that is absent in most other ligand systems.

Organic ligands are obviously represented by a major number of examples. Thus, the structure of the dimethyl sulfoxide (DMSO)-solvated thorium(IV) ions was studied in solution by EXAFS) and the structure of the solid oxonium *bis*[nonakis(κ*O*-dimethyl sulfoxide)]thorium(IV) trifluoromethane-sulfonate dihydrate, (H_3_O)[Th((CH_3_)_2_SO)_9_]_2_(CF_3_SO_3_)_9_·2H_2_O was determined [17]. It consists of two individual nonakis(κ-*O*-dimethyl sulfoxide)thorium(IV) units, both of which have a tricapped trigonal prismatic configuration, as also found earlier in nonakis(dimethyl sulfoxide)thorium(IV) perchlorate. The DMSO-solvated thorium(IV) ion is nine-coordinate in both solution and the solid state with average Th-O bond lengths of 2.45 Å. On the contrary, the dmso-solvated lanthanoid(III) ions are eight-coordinate.

**Figure 2:**
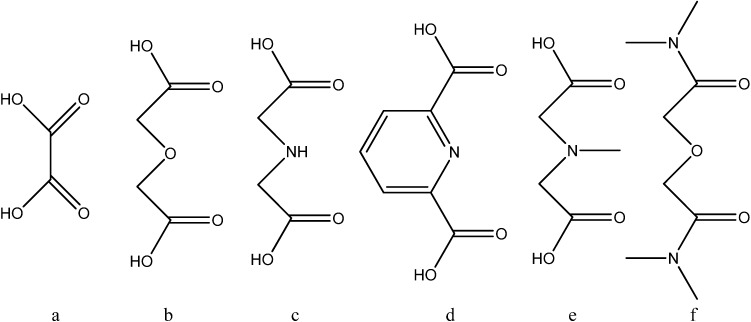
Oxydiamide and dicarboxylic acid ligands: (**a**) oxalic acid (ox), (**b**) oxydiacetic acid (ODA), (**c**) iminodiacetic acid (IDA), (**d**) dipicolinic acid (DPA), (**e**) *N*methyliminodiacetic acid (MIDA), (**f**) *N*,*N*,*N*',*N*'-tetramethyl-3-oxa-glutaramide (TMOGA).

Actinide carboxylates have been extremely widely studied. Thus, the results on the optical absorption and symmetry of the Np(V) complexes with dicarboxylate and diamide ligands (Figure 2) are reviewed [18]. It was demonstrated that the optical absorption properties of the Np(V) complexes are governed by their symmetry. The presence of carboxylates could lead to changes in the forms of actinide ions in solution. For example, hydrated actinide(IV) ions undergo hydrolysis and further polymerization and precipitation with increasing pH [19]. The resulting amorphous and partly crystalline oxydydroxides AnO*_n_*(OH)_4 − 2*n*_·*x*H_2_O can usually be observed as colloids above the An(IV) solubility limit. The aging process of such colloids results in crystalline AnO_2_. The colloids can be avoided in the presence of carboxylates, forming polynuclear complexes in the solution, in a competition in between complexation and hydrolysis. Most of these polynuclear complexes poses a hexanuclear core with general formula [An_6_(*μ*_3_-O)_4_(*μ*_3_-OH)_4_]^12+^ terminated by 12 carboxylate ligands. The An(IV) carboxylates show An-An distances which are ~0.03 Å shorter than the An-An distances in AnO_2_ like colloids. In addition, the complexation of Eu(III), Am(III) and Cm(III) with dicarboxylate anions with O, N or S donor groups was measured in *I* = 6.60 mol/kg (NaClO_4_) at temperatures of 0–60 °C by potentiometry and solvent extraction [20]. It was shown that, despite their endothermic complexation enthalpies, these complexes are stable due to their high complexation entropies.The formation of 1:1:1 ternary complexes of M(EDTA)with the dicarboxylate moiety may favors the formation of several coordination environments of these ternary complex, behaving as bidentate or monodentate coordination modes, depending of the chain length in between both carboxylate coordinating groups (1, for malonate to 4 for adipate).

**Figure 3:**
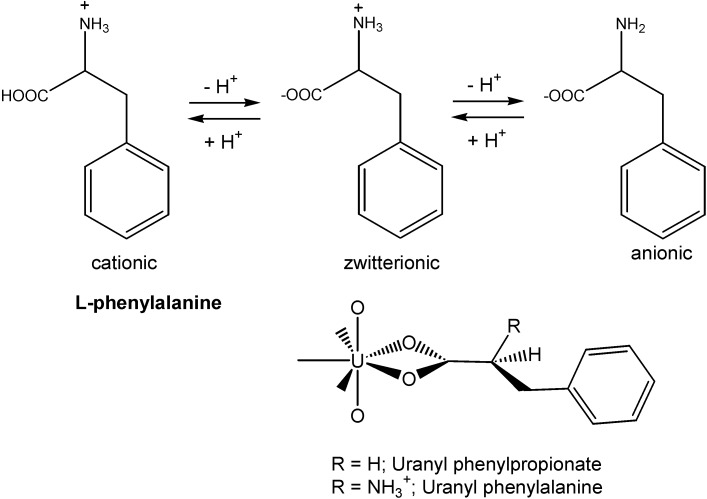
Chemical structures of the ionic forms of l-phenylalanine and of the proposed uranyl(VI) complex.

Among particulate carboxylates, uranyl complexes (Figure 3) with phenylalanine and the analogous ligand phenylpropionate were investigated in aqueous solution by attenuated total reflection (ATR) Fourier transform infrared (FT-IR) spectroscopy [21].

**Figure 4:**
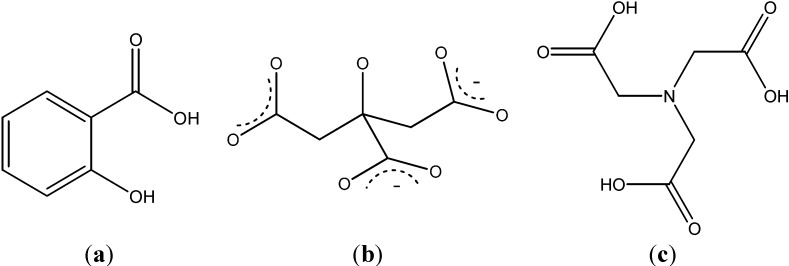
(**a**) Salicylic acid; (**b**) citrate anion, (**c**) nitrilotriacetic acid.

A bidentate binding of the carboxylate group to the actinide ion was observed by the characteristic shifts of the carboxylate modes. The carboxylate functional group was found to be predominant for the binding of the heavy metal ion.Complexes with other organic acids are also common. Thus, the complexation of protactinium(V) by oxalate was studied by a series of methods, indicating the formation of a highly charged anionic complex. The formation constants of PaO(C_2_O_4_)^+^, PaO(C_2_O_4_)_2_^−^, and PaO(C_2_O_4_)_3_^3−^ were determined from solvent extraction data by using protactinium at tracer scale (*C*_Pa_ < 10^−10^ M). Complexation reactions of Pa(V) with oxalate were found to be exothermic with relatively high positive entropic variation [22]. The complexation of americium(III) with salicylic acid (Figure 4a) was studied [23] at trace metal concentrations using a 2.0 m long path flow cell for UV-Vis spectroscopy.

Americium(III) has a very low threshold detection limit of 5 × 10^−9^ M in water at pH 3.0. It was found that at pH 5.0 in an aqueous 0.1 M NaClO_4_ solution, two novel Am(III)-salycylate complexes were formed, as indicated by a red shift of its characteristic absorption band (λ_max_) in the UV-Visible spectra. We would like to note that americium evolves in nuclear power plants and contributes to the activity of radioactive waste, so, it has to be considered in radioactive waste management.

**Figure 5:**
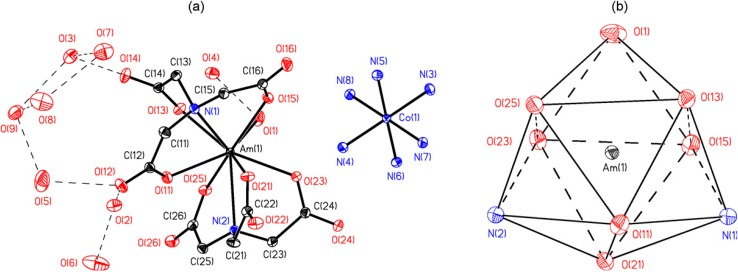
(**a**) ORTEP representation of [Co(NH_3_)_6_][Am(NTA)_2_(H_2_O)]·8H_2_O. Thermal ellipsoids are drawn at the 50% probability level, H atoms omitted for clarity. Dashed lines indicate H-bonding interactions; (**b**) coordination polyhedron of the Am atom showing distorted tricapped trignal prism.

**Figure 6:**
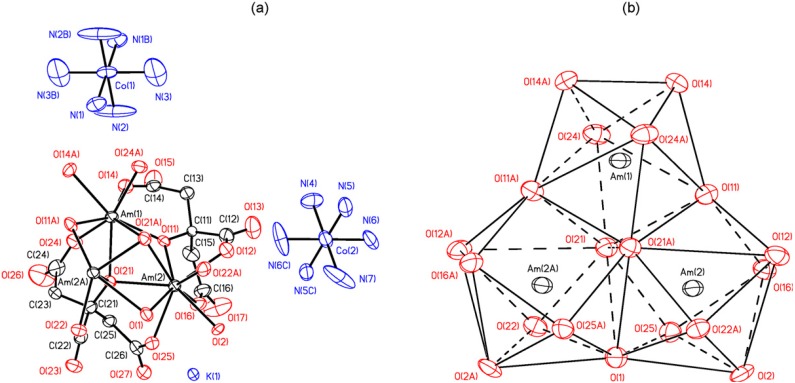
(**a**) ORTEP representation of [Co(NH_3_)_6_]_2_K[Am_3_(Cit)_4_(H_2_O)_3_]·18H_2_O. Thermal ellipsoids are drawn at the 50% probability level, H atoms and coordination water molecules omitted for clarity; (**b**) coordination polyhedra of Am atoms. Symmetry transformations: A – (1 − x, y, 0.5 − z); B – (1 − x, −y, 1 − z); C – (−x, y, 0.5 − z).

Only a few publications concerning the complexation of Am(III) with inorganic and organic ligands are available, especially for the complexation with humic substances [1] and chelating agents. The binary complexation of Am^3+^, Cm^3+^ and Eu^3+^ with citrate anion was studied at I = 6.60 m (NaClO_4_) in the temperatures range of 0–60 °C employing a solvent extraction technique with di-(2-ethylhexyl)phosphoric acid/heptanes [24]. Two complexes, MCit and MCit_2_, were formed at all temperatures. Positive enthalpy and entropy values for the formation of both complexes were interpreted as due to the contributions from the dehydration of the metal ions exceeding the exothermic cation–anion pairing. In addition, two types of ligands that have in common three carboxylic groups, namely the citric acid (citric anion, see Figure 4b) and nitrilotriacetic acid, Figure 4c), and their americium complexes [Co(NH_3_)_6_][Am(NTA)_2_(H_2_O)]·8H_2_O (Figure 5) and [Co(NH_3_)_6_]_2_K[Am_3_(Cit)_4_(H_2_O)_3_]·18H_2_O (Figure 6) were discussed [25]. In all cases the americium complexes were found to be isostructural with their Nd equivalents.

### A.3. Actinide Complexes with N, N, O- and N,S-Containing Ligands

#### A.3.1. Complexes with N-Containing Ligands

**Scheme 1:**
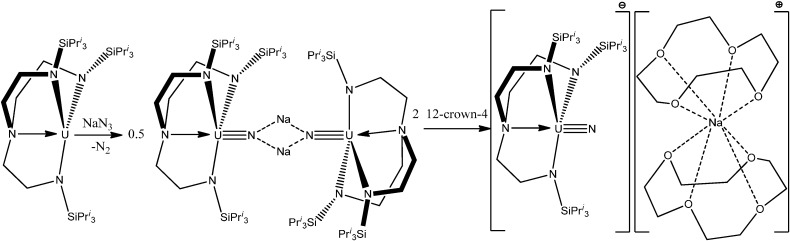
Reaction scheme for formation of [UN(TrenTIPS)][Na(12-crown-4)_2_].

Actinide complexes with N-containing ligands are represented by a variety of examples. Thus, the terminal uranium(V) nitride complex [UN(TrenTIPS)][Na(12-crown-4)_2_] {in which TrenTIPS = [N(CH_2_CH_2_NSiPr*^i^*_3_)_3_]^3−^ and Pr*^i^* = CH(CH_3_)_2_} (Scheme 1) was prepared by reaction of the uranium(III) complex [U(TrenTIPS)] with sodium azide followed by abstraction and encapsulation of the sodium cation by the polydentate crown ether 12-crown-4 [26].

**Scheme 2:**
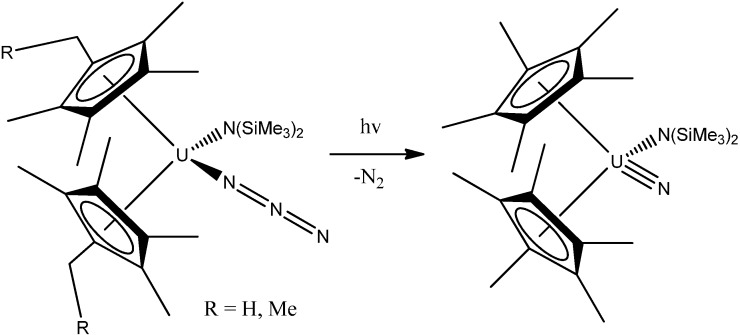
Photolysis of azide complex (left) generates a transient terminal uranium nitride (right).

**Figure 7:**
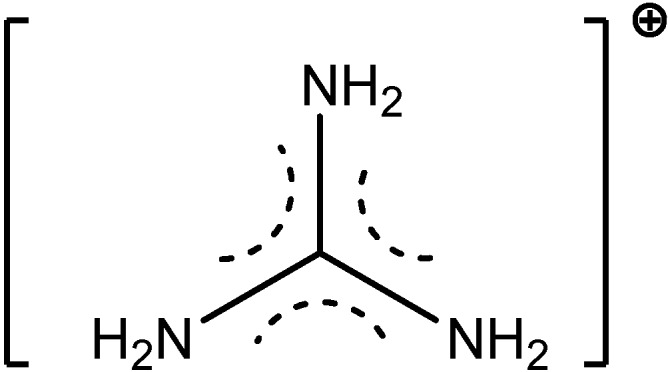
Guanidinium cation.

**Figure 8:**
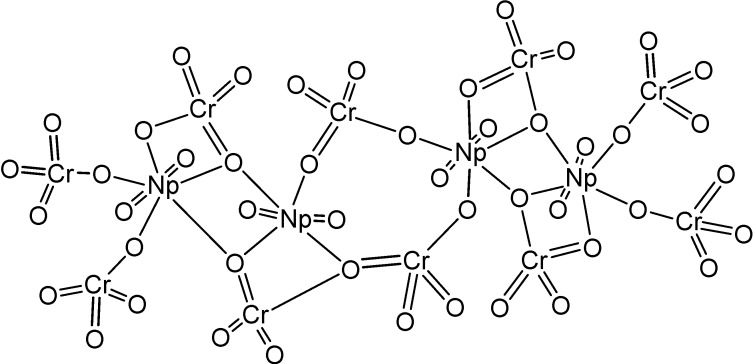
Anionic chain [CpO_2_(CrO_4_)_2_]_n_^3*n*−^ in the structure of [C(NH_2_)_3_]_3_[NpO_2_(CrO_4_)_2_](H_2_O). Thermal vibrational ellipsoids are shown with the 50% probability.

A uranium-terminal nitride bond length of ~1.825 Å was revealed. It should be noted that uranium nitride [U;N]*_x_* compounds may become an interesting alternative nuclear power source, although there is not too much information about their potential use and properties. It was shown [27], that a terminal uranium nitride complex can be generated by photolysis of an azide precursor (Scheme 2). The transient U,N fragment is reactive and undergoes insertion into a ligand C-H bond to generate new N-H and N-C bonds. A complex [C(NH_2_)_3_]_3_[NpO_2_(CrO_4_)_2_](H_2_O) of Np(V) with the chromate ion and an organic outer-sphere guanidinium cation (Figure 7) was isolated from an aqueous solution [28]. Its structure is based on anionic chains [CpO_2_(CrO_4_)_2_]_n_^3*n*−^ (Figure 8) between which exist guanidinium cations and crystallization water molecules. Coordination polyhedra of the Np atoms (pentagonal bipyramids) in the anionic chains are joined in pairs through common equatorial edges.

**Figure 9:**
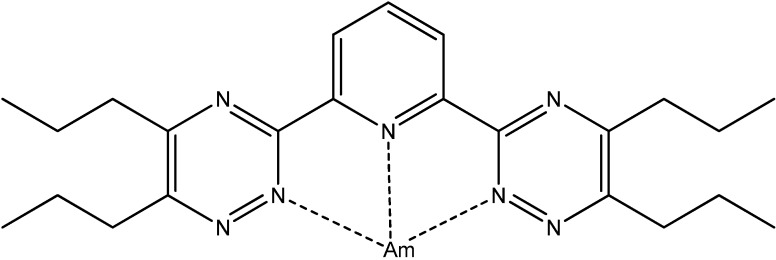
Structure of Am(*n*-PrBTP)_3_^3+^ complex.

5- and 6-member heterocycles containing N-atoms are also known as ligands in actinide complexes. Thus, the selectivity of N-donor containing ligands such as BTPs (alkylated *bis*-triazinylpyridines), for actinide complexation in the presence of lantanides, was investigated [29]. NMR studies of an Am (*n*-PrBTP)_3_^3+^ complex (Figure 9) with a ^15^N labelled ligand showed that it exhibits large differences in 15N chemical shift for coordinating N-atoms in comparison to both lanthanide(III) complexes and the free ligand. The temperature dependence of NMR chemical shifts observed for this complex indicated a weak paramagnetism. On the basis of this fact and the observed large chemical shift for bound nitrogen atoms, the authors concluded that metal–ligand bonding in the reported Am(III) N-donor complex has a larger share of covalence than in lanthanide complexes. Also, the interaction between neptunium(IV) and room-temperature ionic liquids {BmimCl (1-butyl-3-methylimidazolium chloride), BmimMsu (1-butyl-3-methylimidazolium methylsulfate) and BmimSCN (1-butyl-3-methyl-imidazolium thiocyanate)} was studied [30]. They might be useful for the recycling of nuclear fuel and the separation of actinides and fission products from waste solutions.

**Figure 10:**
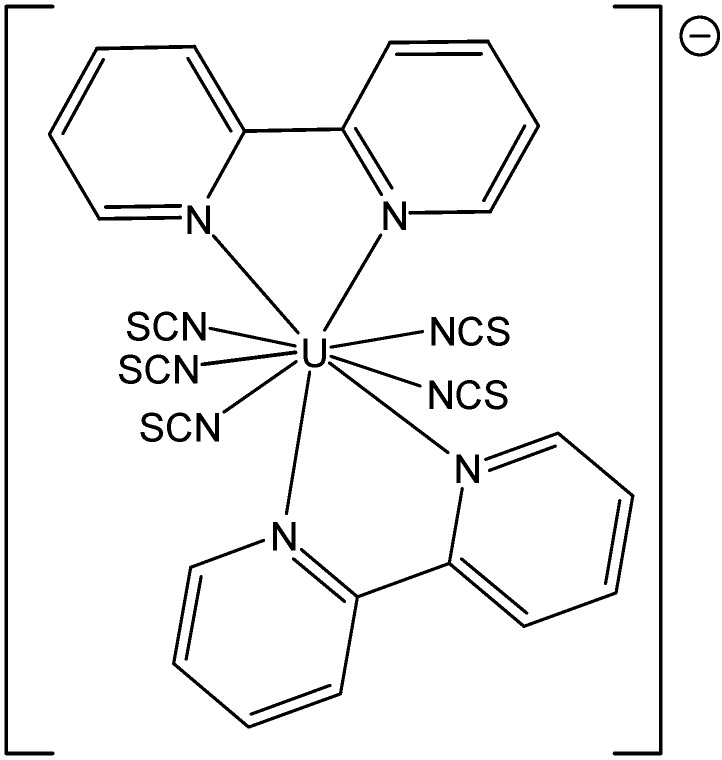
Solid-state structure of [Et_4_N][U(NCS)_5_(bipy)_2_]. Selected average bond lengths (Å): U–N_NCS_ = 2.422; U–N_bipy_ = 2.636; N=C=1.166; C=S=1.618.

Bipyridine adducts are very common [31,32,33,34,35,36] for actinide complexes, as well as in whole in coordination chemistry. As an example, the addition of 2,2'-bipyridine to [U(Tp^Me2^)_2_I] {Tp^Me2^ hydro-*tris*-(3,5-dimethylpyrazolyl)borate ligand} resulted in the displacement of the iodide and the formation of the cationic uranium(III) complex [U(Tp^Me2^)_2_(bipy)]I, isolated as a dark-green solid in good yield [37]. These complexes exhibit a slow relaxation of magnetization (energy barrier of 18.2 cm^−1^), with a T_c_ of 4.5 K with frequency dependent magnetic properties, characteristic of single-molecule-magnet behavior (currently only the third example of a uranium compound with such behavior). Also, the solid-state structure of the known complex [Et_4_N][U(NCS)_5_(bipy)_2_] (Figure 10) was re-determined and a detailed spectroscopic and magnetic study was performed in order to confirm the oxidation states of both metal and bipy ligand [38]. On the basis of electronic absorption, infrared spectroscopy data, emission spectroscopy and variable temperature magnetic measurements it was suggested that the uranium is in its +4 oxidation state. The bipy ligands are neutral, innocent ligands and not, as would be inferred from just a solid state structure, radical anions.

**Scheme 3:**
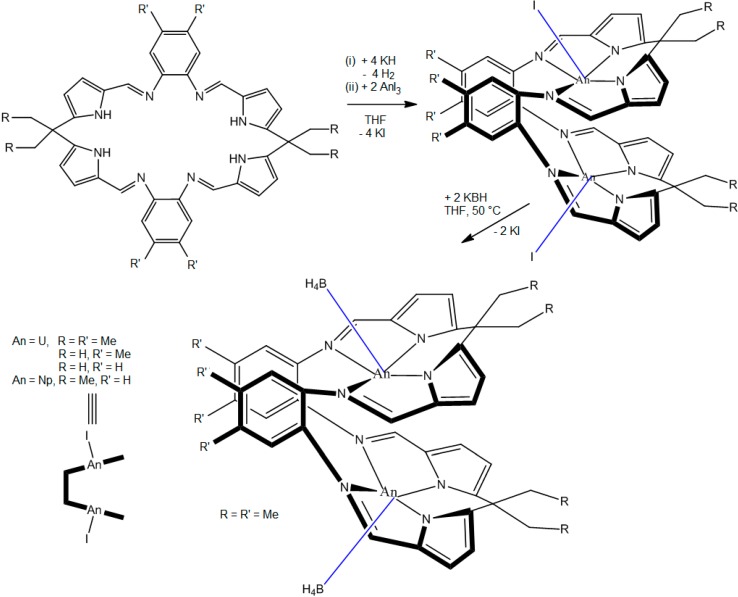
Synthesis of binuclear U^III^ and Np^III^ complexes of the Schiff-base pyrrolic macrocycles H_4_L, H_4_L', and H_4_L''.

A few examples of actinide macrocycle complexes on the basis of N-containing heterocycles are known. Thus, syntheses of the bimetallic uranium(III) and neptunium(III) complexes [(UI)_2_(L)], [(NpI)_2_(L)], and [{U(BH_4_)}_2_(L)] (Scheme 3) of the Schiff-base pyrrole macrocycles L are described [39].

**Scheme 4:**
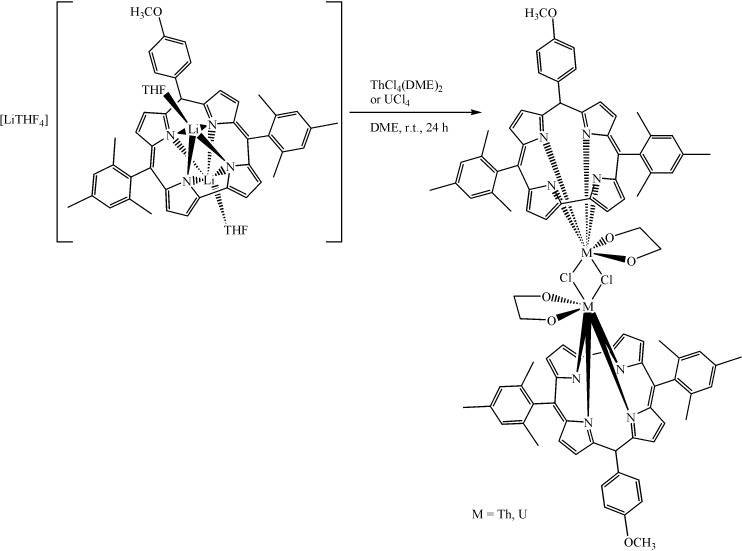
Synthesis of thorium(IV) corrole and uranium(IV) corrole.

Fitting of the variable-temperature solid-state magnetic data allowed the prediction of polymeric structures for these compounds in the solid state. In addition, thorium(IV) and uranium(IV) macrocycles of Mes_2_(*p*-OMePh)corrole were synthesized [40] via salt metathesis (Scheme 4) with the corresponding lithium corrole in remarkably high yields (93% and 83%, respectively). Both complexes are dimeric, having two metal centers bridged via bis(μ-chlorido)linkages. In each case, the corrole ring showed a large distortion from planarity, with the Th(IV) and U(IV) ions residing unusually far (1.403 and 1.330 Å, respectively) from the N_4_ plane of the ligand.

Distinct amines also form a series of complexes with actinide ions. Thus, the extraction of Th(IV) with N-*n*-octylaniline and trioctylamine (TOA) in xylene, from an acid aqueous sulphuric acid was reported [41]. The effects of varying the concentration of sulphuric acid, N-*n*-octylaniline and trioctylamine on the distribution ratio of thorium were studied. Based on the obtained results, the possible extraction mechanism is shown in reactions (1–2). The method can be extended to the analysis of thorium in monazite sand and the gas mantle:

Th^4+^ + 3SO_4_^2−^ ↔ [Th(SO_4_)_3_]^2−^ (1)

mRR′-NH + nR''_3_N + [Th(SO_4_)_3_]^2−^ ↔ mRR′ NH^+^_2_**^.^**Th(SO_4_)_3_^2−**.**^*n* R''_3_NH^+^ (2)

where R = C_6_H_5_ and R' = R'' = C_8_H_17_.

**Figure 11:**
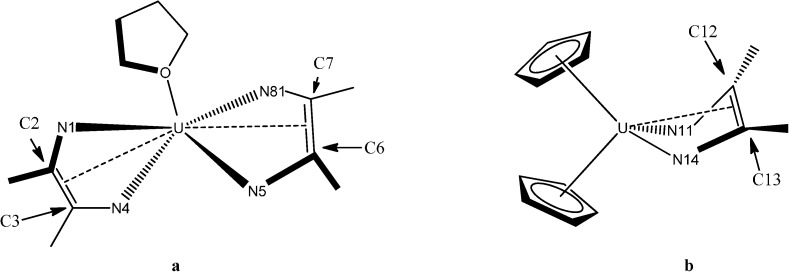
Molecules used for computational study. Aryl groups have been omitted for clarity.

The electronic structures of two uranium compounds supported by redox-active α-diimine ligands, (^Mes^DAB^Me^)_2_U(THF) (Figure 11a) and Cp_2_U(^Mes^DAB^Me^) (Figure 11b) (^Mes^DAB^Me^ = [ArN=C(Me)C(Me)=NAr]; Ar = 2,4,6-trimethylphenyl (Mes)), were investigated [42] using both density functional theory and multiconfigurational selfconsistent field methods. It was established that both uranium centers are tetravalent, that the ligands are reduced by two electrons, and that the ground states of these molecules are triplets. Energetically low-lying singlet states are accessible, and some transitions to these states are visible in the electronic absorption spectrum. The computational analysis presented supports the reduction of all α-diimine ligands in these compounds by two electrons, which was demonstrated experimentally.

**Scheme 5:**
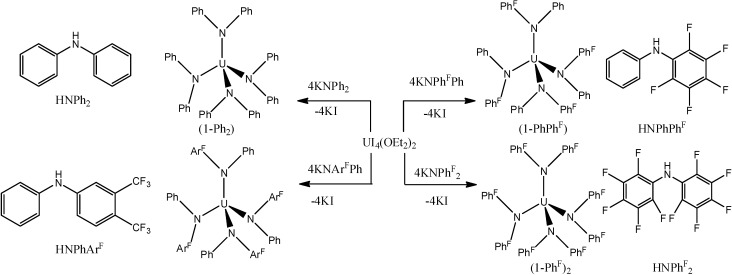
Synthesis of uranium(IV) diarylamide complexes from UI_4_(Et_2_O)_2_.

Finally, the fluorinated diarylamines HNPhPh^F^, HNPh^F^_2_, HNPhAr^F^, Ph^F^ = 2,3,4,5,6-pentafluoro-phenyl, Ar^F^ = 3,5-*bis*(trifluoromethyl)phenyl, were used to prepare homoleptic complexes of uranium(III, IV) ions from UI_4_(Et_2_O)_2_ (Scheme 5) [43]. Despite being electronpoor amines with little steric bulk, their coordinated amide ligands exhibited direct control over the coordination environment through a subtle, cooperative interplay of multiple labile F/U dative interactions and favorable arene–arene interactions.

**Figure 12:**
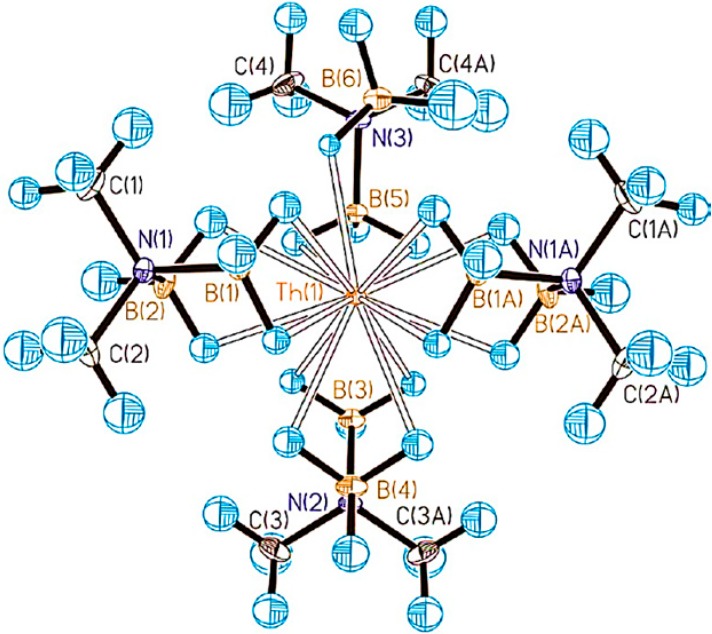
Molecular structure of [Th(H_3_BNMe_2_BH_3_)_4_] from neutron diffraction data. Ellipsoids are drawn at the 20% probability level. Th orange, B tan, N purple, C black, H blue.

Containing extremely large metal atoms, actinide complexes could have unusual structural characteristics. Thus, the synthesis and studies of the first 15-coordinate complex (Figure 12) was reported [44].

Reaction of ThCl_4_ with four equivalents of sodium N,N-dimethylaminodiboranate, Na(H_3_BNMe_2_BH_3_), in THF produced [Th(H_3_BNMe_2_BH_3_)_4_], which could be isolated as colorless prisms by crystallization from diethyl ether. DFT calculations suggested that this complex may adopt a 16-coordinate structure in the gas phase. The isolated molecule has full *D_2d_* symmetry with a coordination number of 16, but that the crowded nature of the inner coordination sphere is sufficiently destabilizing that molecule distorts and becomes 15-coordinate in the solid state. This is the hightest Werner coordination number for a metal complex reported to the date, and was made possible by combining a very large metal atom with very small ligands.

#### A.3.2. Actinide Complexes with N,O- and N,S-Containing Ligands

**Figure 13:**
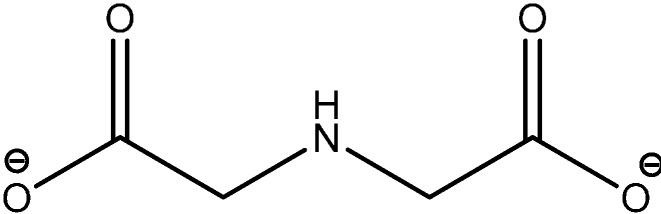
IDA (iminodiacetate).

A series of N,O- and some N,S-containing ligands are represented by a series of Schiff bases, iminoacetates and other amino/amido/imino derivatives, among others. Thus, the stability and the associated thermodynamic parameters of the binary and the ternary complexes of trivalent Am and Cm with iminodiacetate (IDA, Figure 13) and with EDTA+IDA, were determined by using a solvent extraction technique for aqueous solutions of I = 6.60 m (NaClO_4_) at temperatures of 0–60 °C [45]. The endothermic enthalpy and the positive entropy reflected the significant effect of dehydration in the formation of these complexes at high ionic strength.

**Figure 14:**
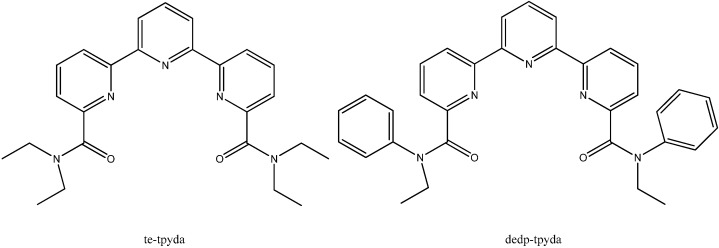
Structure of tpy ligands.

Functionalized bitopic terpyridine(tpy)-*diamide* N,O-ligands (Figure 14) were recently developed for the group actinide separation by solvent extraction. In order to acquire a better understanding of their coordination mode in solution, the protonation and the formation of Am(III) and U(VI) complexes with bitopic N,O- containing ligands in methanol/water homogeneous mixtures was studied [46]. When the terpyridine moiety contained amide functional groups, the extracting properties of these ligands was improved, due tochanges in their basicity. Two predominant inner-sphere coordination modes were found from the DFT calculations: one mode where the cation is coordinated by the nitrogen atoms of the cavity and by the amide oxygen atoms and the other mode where the cation is only coordinated by the two amide oxygen atoms and by solvent molecules.

**Scheme 6:**
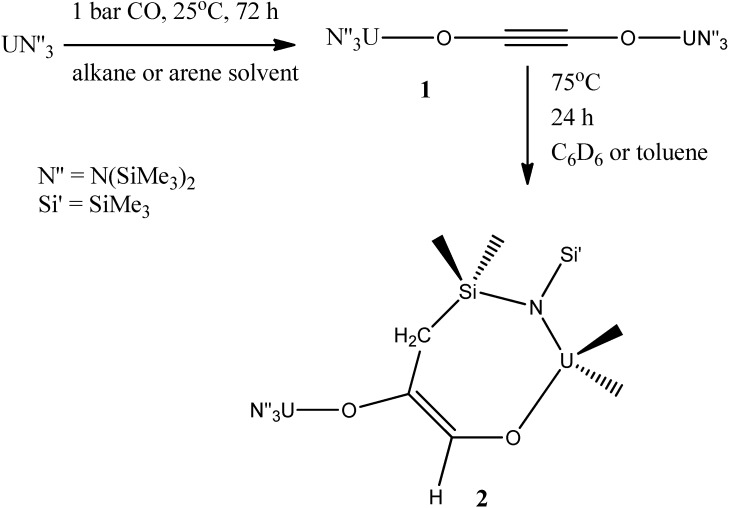
Coupling and functionalization of carbon monoxide by the trivalenturanium amide to form a uranium-coordinated ynediolate **1**, and then an ene-diolate **2**.

Also, it was demonstrated that an uranium(III) *tris*(amide) complex was capable to selectively couple CO into a linear ynediolate [OCCO]^2−^ dianion, at ambient conditions (room temperature and atmospheric pressure), in catalytic concentrations (Scheme 6) [47].

**Scheme 7:**
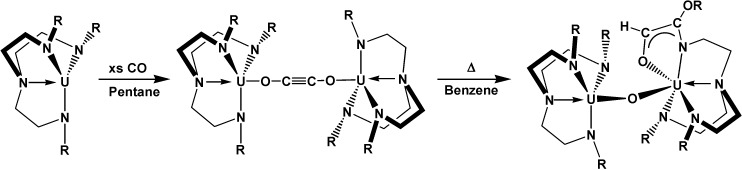
Reaction of CO with uranium complex (R = SiMe_2_Bu*^t^*).

This compound was able, warming the mixture, to activate a C-H bond of a methyl group across the CC triple bond, forming a new CC bond and generating a functionalized enediolate dianion. As a great contribution of this research for the area of reductive activation reactions of small, traditionally inert molecules such as dinitrogen and carbon dioxide, demonstrated for trivalent uranium complexes, the observed ready interconversion between the U^III^ and U^IV^ oxidation states suggested that catalytic systems based on this coupling and functionaliation are viable. It is notable that the reaction occurs with such a simple coordination compound—an amide that is made from simple commercially available ligands (the precursor amide salt currently costs under €100 per mol). In addition, under mild conditions a simple triamidoamine uranium(III) complex (Scheme 7) can reductively homologate CO and be recycled for reuse [48].

**Figure 15:**
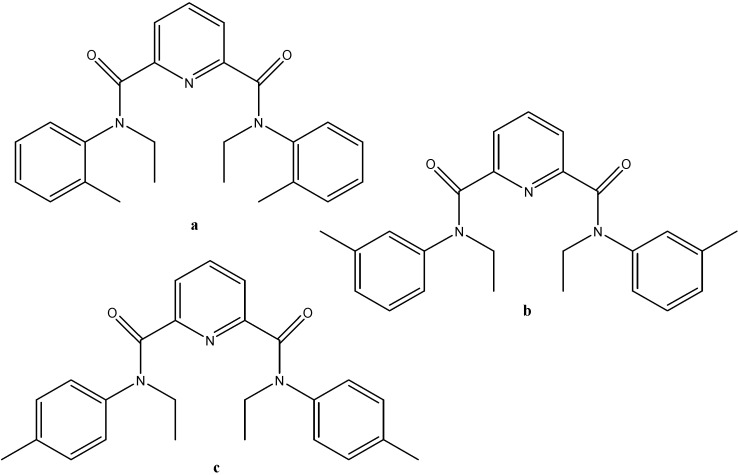
Structures of (**a**) Et(o)TDPA, (**b**) Et(m)TDPA, (**c**) Et(p)TDPA.

Structural analyses of uranyl complexes with isomers of *N*,*N'*-diethyl-*N*,*N'*-ditolyldipicolinamide (EtTDPA, Figure 15) were carried out using IR spectroscopy and single crystal X-ray diffraction [49]. From these analyses, it was determined that complexation takes place through coordination with the carbonyl and pyridine nitrogen moieties.

The uranyl complexes showed space groups of *Pbca* for Et(p)TDPA and *P*21*/n* for Et(o)TDPA. Also, the magnetic properties of the triangular molecular nanomagnet [UO_2_L]_3_ {L = 2-(4-tolyl)-1,3-*bis*(quinolyl)malondiiminate} were investigated through electron paramagnetic resonance spectroscopy, high-field magnetization and susceptibility measurements [50]. The results showed that [UO_2_L]_3_ has a non-magnetic groud state (doublet) due to the chiral arrangement of the uranium magnetic moments to two opposite positions. Quantum tunneling of the non-collinear magnetization, in the presence of a perpendicular external magnetic field results explains its non-axial character of the single-ion crystal field.

**Figure 16:**
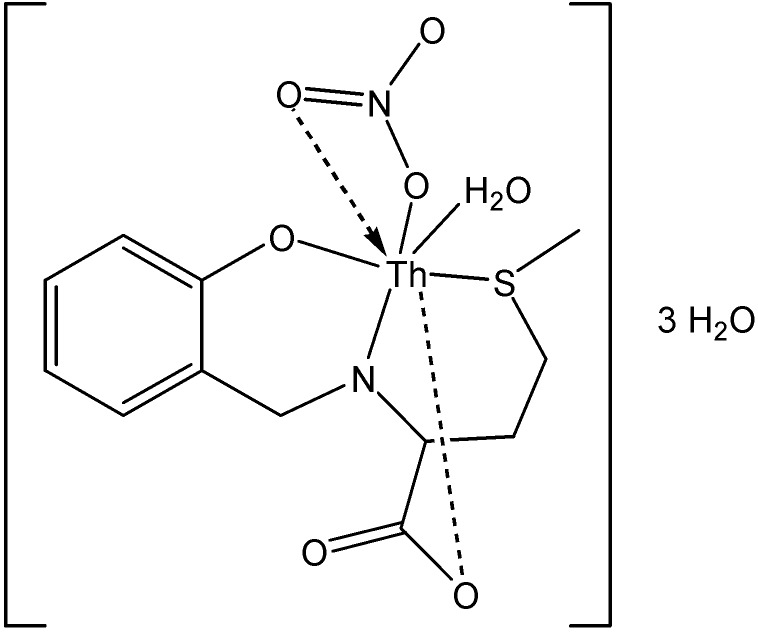
Structure of the suggested metal complex.

**Figure 17:**
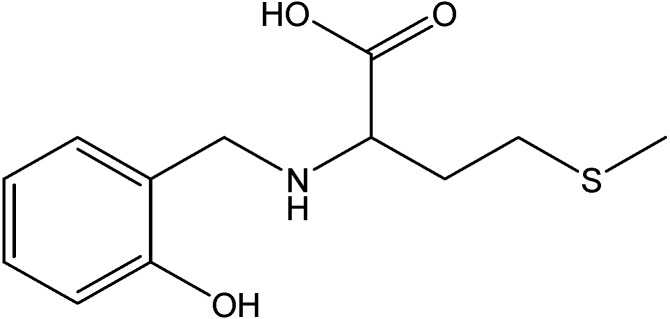
2-(2-Hydroxybenzylamino)-4-(methylthio)butanoic acid.

Metal complex (Figure 16) of Th(IV), with the amino Schiff base ligand, [N-(2-hydroxybenzyl)-L-methionine acid] (H_3_L, Figure 17), was prepared in the presence of triethylamine as a deprotonating agent [51]. The data from thermogravimetricanalysis clearly indicated that its decompositionproceeds in four or five steps and theorganic part decomposed in one or twointermediates. The decomposition of the complex ended with metal oxide and carbon residue.

**Figure 18:**
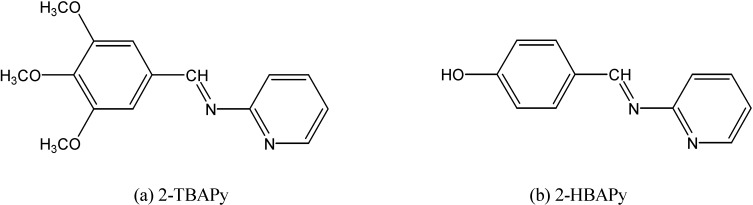
(**a**) 2*N*-[3,4,5-trimethoxybenzylidene]aminopyridine and (**b**) 2*N*-[4-hydroxybenzylidene]aminopyridine.

**Figure 19:**
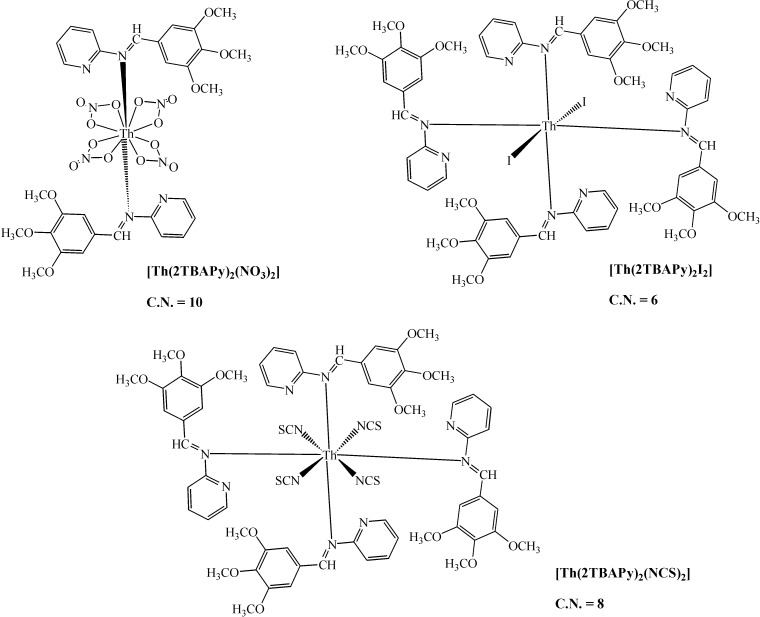
The proposed coordination numbers and structures for selected thorium complexes of 2-TBAPy and 2-HBAPy.

The Schiff bases and their complexes were screened for their antibacterial (*E. coli*, *Staphylococcus aureus*) and antifungal (*Aspergillus flavus* and *Candida albicans*) activities. Several Th(IV) and [UO_2_]^+2^ complexes with Schiff base ligands prepared from *p*-trimethoxybenzaldehyde, *p*-hydroxy-benzaldehyde and 2-aminopyridine (Figure 18) were prepared and their structures and physical and chemical properties reported (Figure 19) [52].

**Figure 20:**
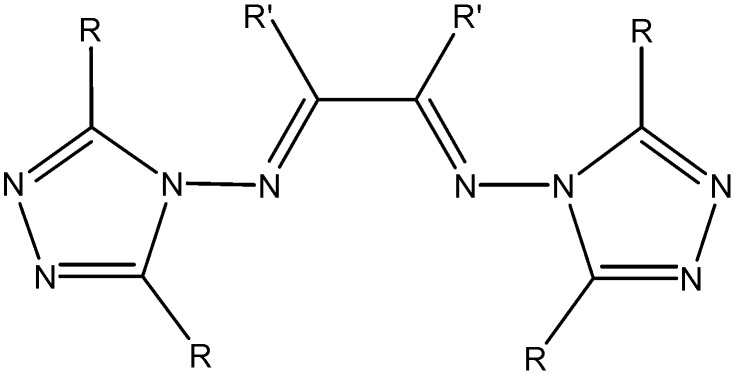
Schiff bases derived from 3-substituted-4-amino-5-mercapto-1,2,4-triazole.

**Figure 21:**
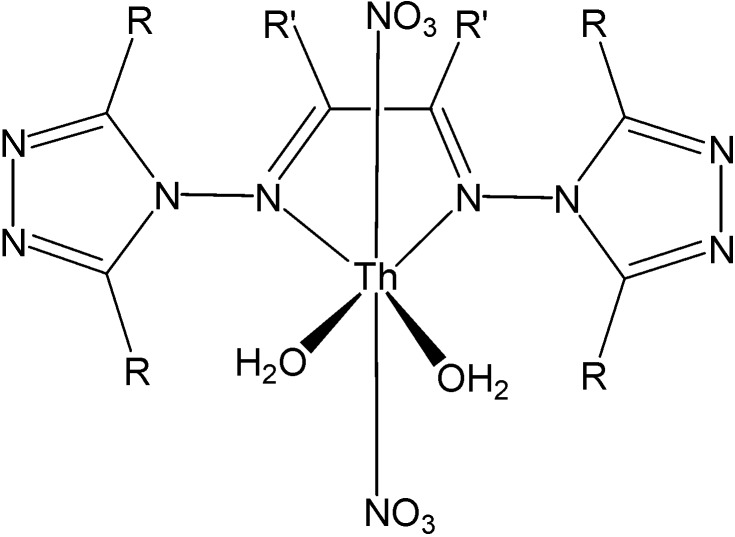
Proposed structure of thorium Schiff-base complexes.

These complexes present a wide range of coordination numbers (from 6 to 10), and some of them have antibacterial and antifungal action. In addition, light yellow thorium(IV) six-coordinate complexes were synthesized by reacting Th(IV) nitrate with Schiff bases (Figure 20) derived from 3-substituted-4-amino-5-mercapto-1,2,4-triazole and glyoxal/biacetyl/ benzyl in ethanol [53]. All these complexes are insoluble in DMF and DMSO. The involvement of both C=N groups in the complex formation was suggested, keeping SH groups away from the coordination (Figure 21).

**Figure 22:**
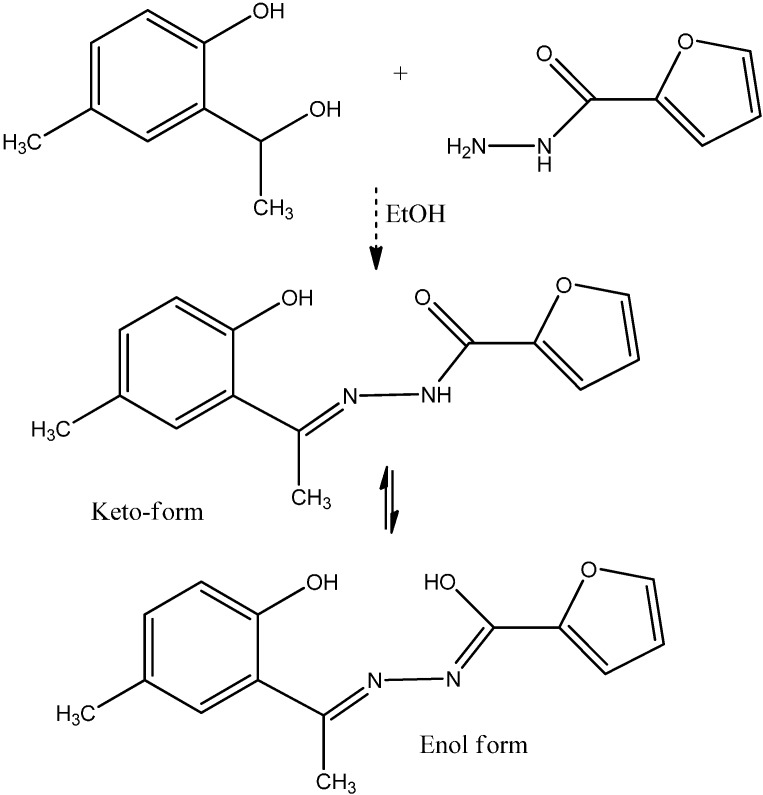
Hydrazone ligand derived from 2-hydroxy-5-methylacetophenone and 2-furoic acid hydrazide.

Dioxouranium(VI) and thorium(IV) complexes of ONO-hydrazone ligand derived from 2-hydroxy-5-methylacetophenone and 2-furoic acid hydrazide (Figure 22) were synthesized and characterized [54].

**Figure 23:**
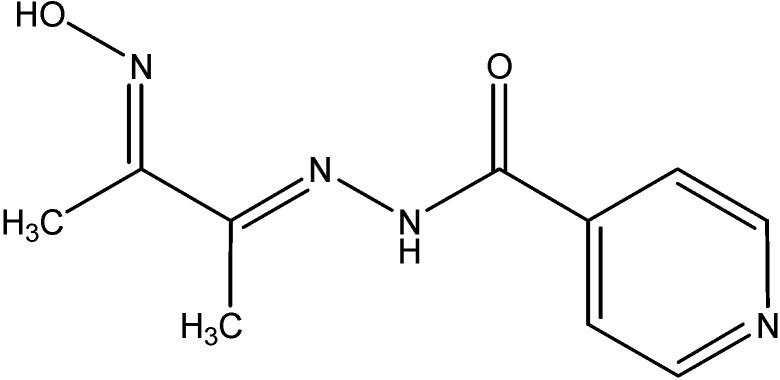
Structure of diacetyl monoxime isonicotinoyl hydrazone (DMIH).

The compounds show semiconductingbehavior as their conductivity increases with increasing temperature. The ligand and its complexes have also been screened for their antibacterial and antifungal activities. The isolated complexes are bright in color, quite air stable, can be stored for long periods, insoluble in water, soluble to very limited extent in common organic solvents but to a considerable extent in DMF and DMSO. Other hydrazone complexes also possess useful applications. For instance, thorium(IV) forms a yellow colored water soluble complex with diacetyl monoxime isonicotinoyl hydrazone reagent DMIH (Figure 23) in acidic buffer of pH 5.0 with λ_max_ at 352 nm [55]. This simple method using DMIH as a spectrophotometric reagent can be applied for the determination of thorium(IV) in aqueous medium.

**Figure 24:**
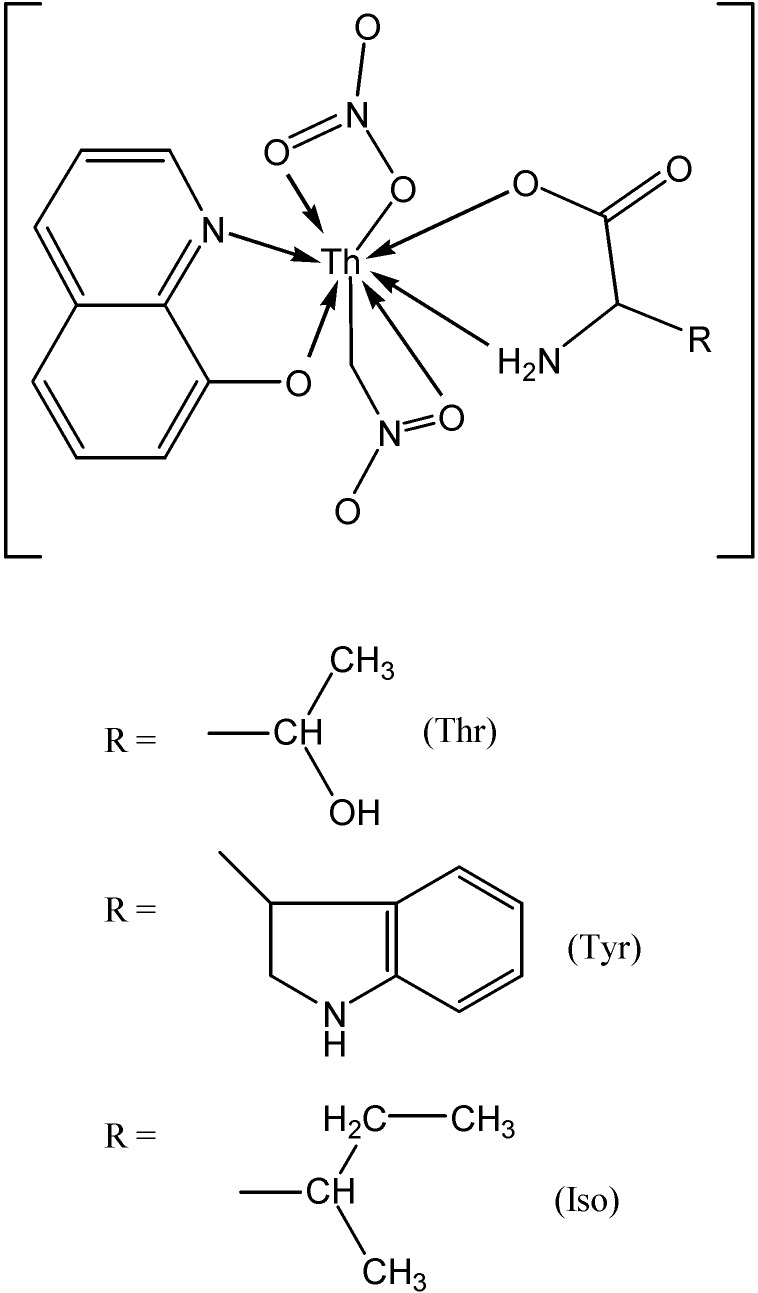
Proposed structures and bonding for the 8-hydroxyquinoline complexes.

Mixed-ligand diamagnetic Th(IV) complexes (Figure 24) of the type [M(Q)(L)(NO_3_)_2_]**^.^**2H_2_O were synthesized [56] using 8-hydroxyquinoline as a primary ligand and N- and/or O-donor amino acids such as L-threonine, L-tryptophan and L-isoleucine as secondary ligands.

**Figure 25:**
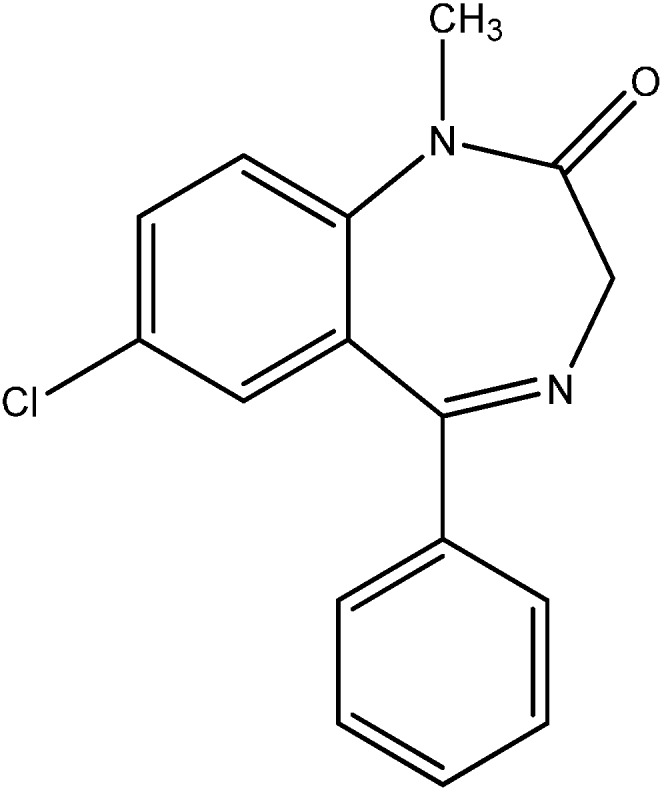
Diazepam [7-chloro-1-methyl-5-phenyl-3H-1,4-benzodiazepine-2-one].

The electrical conductance studies of the complexes in DMF in 10^−3^ M concentration indicate their non-electrolytic nature. Bonding of the metal ion takes place through N- and O-donor atoms of the ligands. In addition, complexes of diazepam (DZM, Figure 25) with the metal nitrates of thorium and uranium were synthesized [57]. The complexes were found to have the general composition [L(M^1^)(NO_3_)_4_] and [L(M^2^)(NO_3_)_2_], where L= ligand (DZM), M^1^ = Th(IV) and M^2^ = UO_2_(II). The complexes were proposed to be octahedral in geometry. The ligand and its metal complexes were screened for their antimicrobial activities on bacteria (*E. coli*, *S. typhi*, *B. subtilis* and *S. aureus*) and fungi (*A. niger*, *A. flavous*, *P. triticena* and *F. species*). Among other recently reported actinide complexes, we note those with amido/amino phenol ligands [58,59] and containing both sulfonate and carboxylate groups [60] .

### A.4. Actinide Complexes with Calixarenes

**Figure 26:**
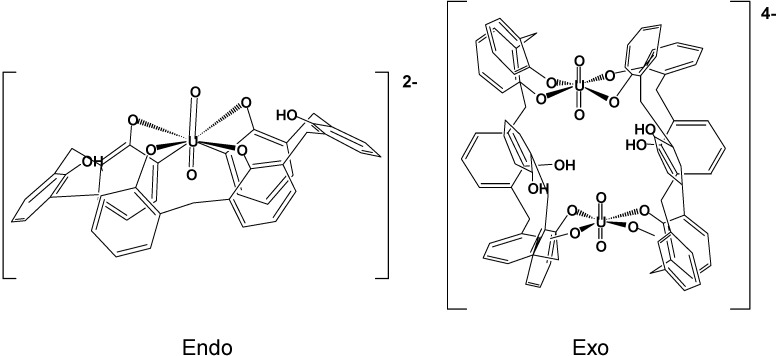
Endo- and exocavity binding in uranyl-calix[6]arene complexes.

**Scheme 8:**
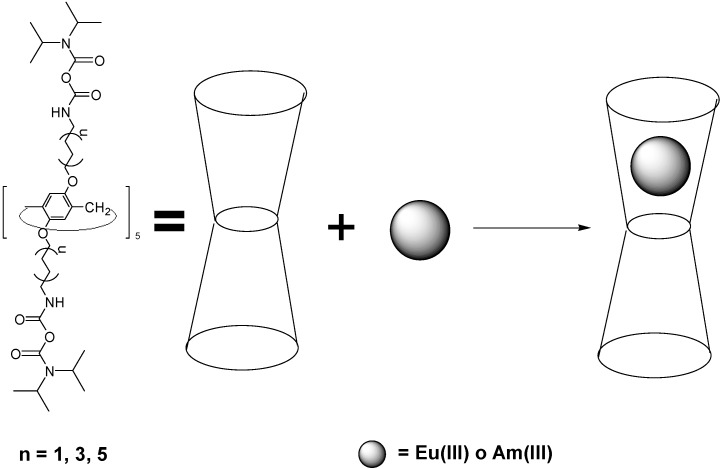
The extraction of trivalent Am(III) and Eu(III) cations with three novel pillar[5]arenes substituted by ten diglycolamide groups (P5DGAs).

Calixarenes (in particular phosphinoylated calixarenes as *p*-*tert*-butylcalix[4]arene, forming stable thorium complexes with 1:1 and 1:2 stoichiometries in organic media [61], or calixarene-based picolinamides and malonamides [62]) feature high coordination ability toward *f* elements and a great potential for actinide/rare earth separation. In particular, they are applied as as macrocyclic ligands for uranium(VI) [63], showing endo- and exocavity binding in uranyl-calix[6]arene complexes (Figure 26). Calixarene complexes are mainly used for analytical or extraction/separation purposes. Thus, a new class of calixarene analogues, pillar[5]arenes, having ten diglycolamide (DGA) pendant groups as arms on both rims of the pillar structure, were prepared and their affinity toward Am(III) and Eu(III) evaluated, as potential novel chelating agents for rare-earth and actinide extraction (Scheme 8) [64].

**Figure 27:**
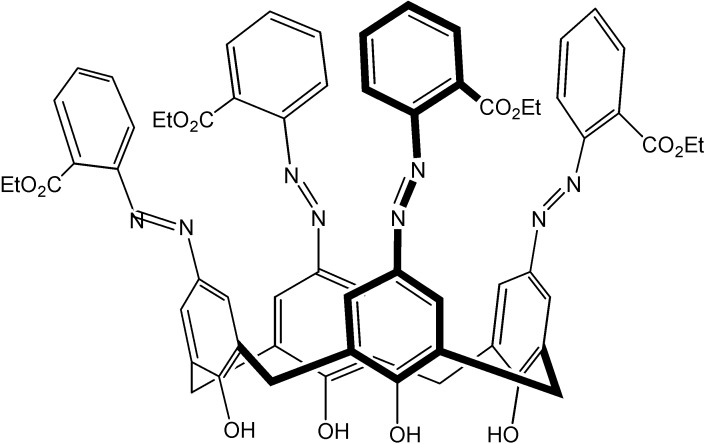
Structure of TEAC.

These extractants exhibited excellent separation and extraction efficiency, suggesting its significant potential for nuclear waste remediation. Laser induced fluorescence experiments disclosed strong complexation of the trivalent metal ions with the pillararene-DGA ligands. As a new class of extractants with a framework of pillar conformation that is quite different from the calixarene extractants, pillararene-based diglycolamides may hold potential for the efficient separation of Eu(III) and Am(III) from radioactive liquid nuclear waste. In addition, application of azocalixarene for evaluation of thorium content based on the complex of *o*-ester tetraazophenylcalix[4]arene (TEAC, Figure 27) with thorium(IV) in acetate buffer solution was offered [65]. This recommended method could be applied for determination of thorium concentration in some monazite ore with high confident results.

### A.5. Actinide Complexes with P-Containing Ligands

**Figure 28:**
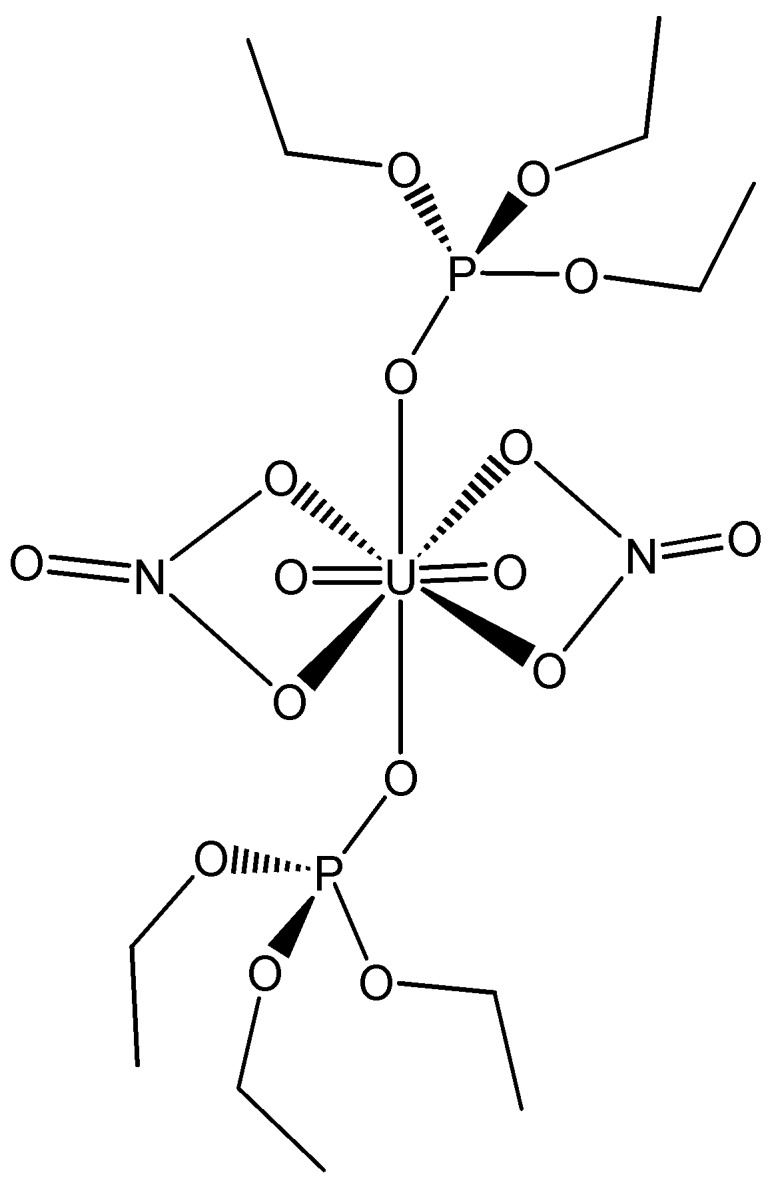
Schematic diagram of complex structure UO_2_(NO_3_)_2_·2TEP.

A few P-containing actinide complexes have been recently reported with classic P-ligands. Thus, (Ph_4_P)_2_UO_2_I_4_**^.^**2NCCH_3_ was prepared [66] according to the reaction (3). The redcrystals were soluble in MeCN, but decomposed quickly insolvents such as methanol or THF. It was noted that, whereas UO_2_I_2_**^.^***x*H_2_O is thermally unstable in the solid state at room temperature, the neutral UO_2_I_2_{OP(NMe_2_)_3_}_2_, UO_2_I_2_(OPPh_3_)_2_, and UO_2_I_2_(py)_3_, as well as (Ph_4_P)_2_UO_2_I_4_**^.^**2NCCH_3_, are all stable in the solid state at r.t. Extraction of Am(III) and Cm(III) [67], as well as Np(VI) [68], between tri-*n*-butyl phosphate solution and molten calcium nitrate hydrate Ca(NO_3_)_2_**^.^**RH_2_O was investigated radiochemically. The extraction reaction of Am and Cm in the Ca(NO_3_)_2_·RH_2_O-TBP system is considered to be the same as the reaction in the HNO_3_-TBP system (4). The distribution ratio was found to be inversely related to the water activity (in the range of water content *R* = 3.5–8.0). This dependence in the hydrate melt changes according to log*a*_H2O_ = −0.4, which corresponds to *R* = 5.0. The distribution of Np(IV) between 0.08–4.5 M HNO_3(aq,eqm)_ and ~30% tri-*n*-butyl phosphate was modelled, accounting for the formation of 1:1 and 1:2 nitrate complexes and Np(IV) hydrolysis in the aqueous phase and the extraction of Np(NO_3_)_4_(TBP)_2_ into TBP [69]. In addition, the role of water in the formation of associates from nanosized complexes of uranium in a supercritical carbon dioxide (SC CO_2_) medium was studied [70]. It was found experimentally that water in the SC CO_2_ exists in the form of microdrops and at a pressure of 10 MPa and a temperature of 40 °C the UO_2_(NO_3_)_2_·2(C_4_H_9_O)_3_PO (TEP) complex (Figure 28) may take on hydrophilic properties. The complex above would concentrate in water microdrops, and its concentration in water microdrops gives rise to the formation of associates, the size of which was determined by microdrop dimensions.

UO_2_I_2_**^.^***x*H_2_O + 2Ph_4_PI →(Ph_4_P)_2_UO_2_I_4_ + *x*H_2_O (3)

M(H_2_O)*_n_*^3+^ + 3NO_3_^−^+ 3TBP → M(NO_3_)_3_ 3TBP + *n*H_2_O (4)

where M indicates Am or Cm and *n* indicates the hydration number.

### A.6. Actinide Organometallic Complexes

**Scheme 9:**
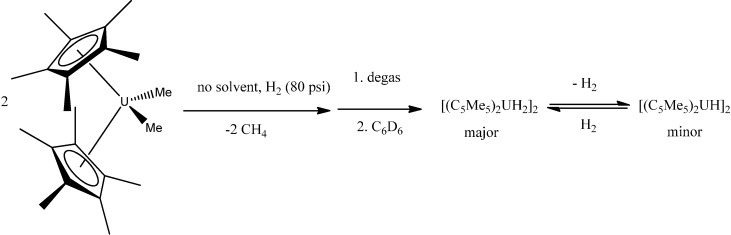
Reaction of solid (C_5_Me_5_)_2_UMe_2_ with H_2_ gas.

**Scheme 10:**
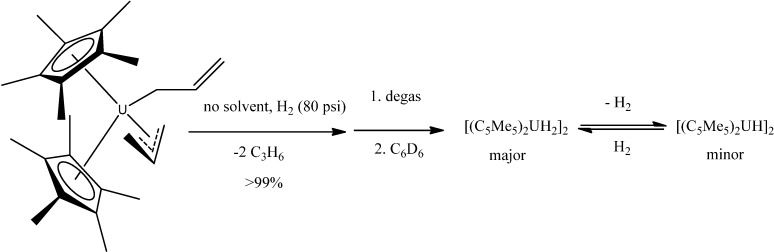
Reaction of solid (C_5_Me_5_)_2_U(C_3_H_5_)_2_ with H_2_ gas.

**Scheme 11:**
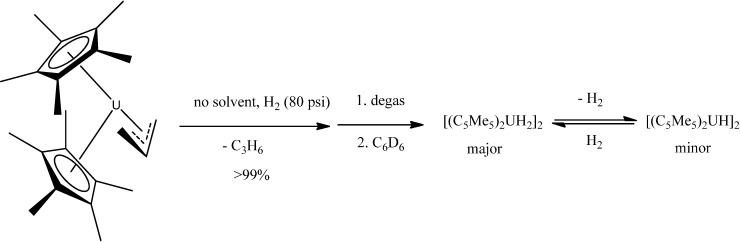
Reaction of solid (C_5_Me_5_)_2_U(C_3_H_5_) with H_2_ gas.

A series of actinide organometallics is known, mainly metallocene-like complexes of uranium and thorium {although the arene-bridged complex (μ-toluene)U_2_(N[*t*Bu]Ar)_4_ (Ar = 3,5-C_6_H_3_Me_2_) is also known [71] }. Thus, gas/solid reactions involving H_2_ and CO_2_ with the metallocenes (C_5_Me_5_)_2_UMe_2_ and (C_5_Me_5_)_2_U(allyl)_2_ as solids in the absence of solvent provided an improved method to make organouranium hydride and carboxylate products (Scheme 9, Scheme 10 and Scheme 11) [72].

The reaction of CO_2_ (80 psi) with solid (C_5_Me_5_)_2_UMe_2_ forms the monocarboxylate (C_5_Me_5_)_2_U(O_2_CCH_3_-*κ^2^O*,*O*')Me, in contrast to the solution reaction that forms the diacetate (C_5_Me_5_)_2_U(O_2_CCH_3_-*κ^2^O*,*O*')_2_ in minutes.

**Scheme 12:**
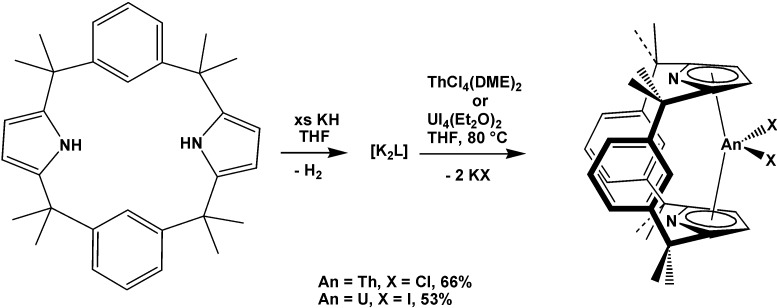
Synthesis of [ThCl_2_(L)] and [UI_2_(L)].

The bipyridyl thorium metallocenes [η^5^-1,2,4-(Me_3_C)_3_C_5_H_2_]_2_Th(bipy) and [η^5^-1,3-(Me_3_C)_2_C_5_H_3_]_2_Th(bipy) (see also bipy complexes above) were investigated by magnetic susceptibility and computational studies [73]. It was revealed that these complexes are not diamagnetic, but they behave as temperature independent paramagnets. In addition, they react with Ph_2_CS to give [η^5^-1,2,4-(Me_3_C)_3_C_5_H_2_]_2_Th[(bipy)(SCPh_2_)] and [η^5^-1,3-(Me_3_C)_2_C_5_H_3_]_2_Th[(bipy)(SCPh_2_)], respectively, in quantitative conversions. Also, conformationally restricted Th^IV^ and U^IV^ complexes, [ThCl_2_(L)] and [UI_2_(L)] (Scheme 12), of the small-cavity, dipyrrolide, dianionic macrocycle *trans*-calix[2]benzene[2]pyrrolide (L) (see also calixarene complexes above) were reported and were shown to have unusual k^5^:k^5^ binding in a bent metallocene-type structure [74].

**Scheme 13:**
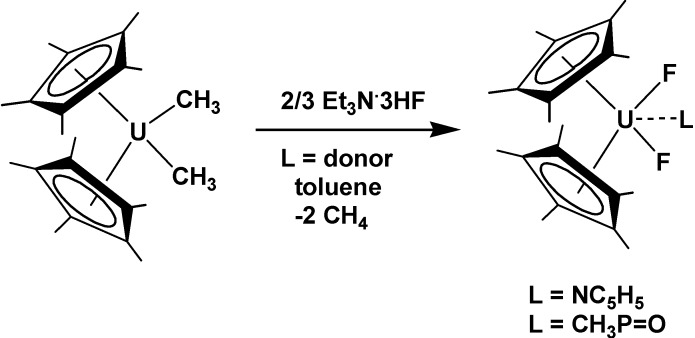
Formation of uranium(IV) difluoride complexes (C_5_Me_5_)_2_UF_2_(L).

Reaction of the uranium alkyl complex (C_5_Me_5_)_2_UMe_2_ (Scheme 13) with Et_3_N·3HF in toluene in the presence of a donor ligand (pyridine or trimethylphosphine oxide) resulted in gas evolution and the formation of the uranium(IV) difluoride complexes (C_5_Me_5_)_2_UF_2_(L) (L = NC_5_H_5_, Me_3_P=O) [75]. The fluoride complex (C_5_Me_5_)_2_UF_2_(NC_5_H_5_) was reactive against several trimethylsilyl compounds, showing that the U-F bond may provide of a new synthetic tool for the preparation of new functional groups, presently not available from alkoxide and chloride complexes.

Recently, a new class of bitopic ligands containing phenantroline and 1,3,5-triazine cores and functionalized with picolinamide groups were prepared. The ligands were able to extract and separate actinides selectively at different oxidation states [76].

## Part B. Technetium-99m Chemistry

### B.1. General Concepts on Technetium Complexes

Technetium (Tc) has no stable isotopes; every form of Tc is radioactive. Due to that, there is not almost any natural Tc present on Earth (with the exception of that produced from the spontaneous fission product in uranium ores) and most of it has to be produced synthetically. It was first discovered in 1937 by Carlo Perrier and Emilio Segrè at the University of Palermo by proving that the radioactivity in a molybdenum foil discarded from a cyclotron at Lawrence Berkeley National Laboratory was produced by an element with Z = 43. Technetium short-lived metastable nuclide ^99m^Tc (T_1/2_ 6.015 h, γ-irradiator) was later isolated by Segrè and Glean T. Seaborg at Berkeley and it has been widely used until today as a radiotracer in Nuclear Medicine.

In the last 10 years, around 1,000 papers have been published, reporting the preparation and structural characterization of Tc complexes with a wide variety of ligands, or the use of ^99m^Tc as a radiotracer or radio-emitter in nuclear medicine. Specific applications have been reported in bone scanning, selective imaging of heart, brain, kidney, liver, lungs and other organs, as well as a radiolabeling agent for tumor tissues. Due to its low γ radiation energy (140 keV), short half-life and accessibility, ^99m^Tc has been the most obvious choice in diagnostic nuclear medicine. Some relatively recent reviews on the state-of-the-art of Tc based diagnostic imaging agents, radiotracers and radiopharmaceuticals [77,78,79,80,81,82,83]. The [Tc(CO)_3_]^+^ moiety has been widely exploited for the preparation of bioorganometallic compounds for radiopharmacy and the development of *in vivo* imaging agents [84].Macrocyclic chelating ligands, such as crown ethers have been also reviewed as they may become useful as radiopharmaceuticals for heart imaging [85].

**Figure 29:**
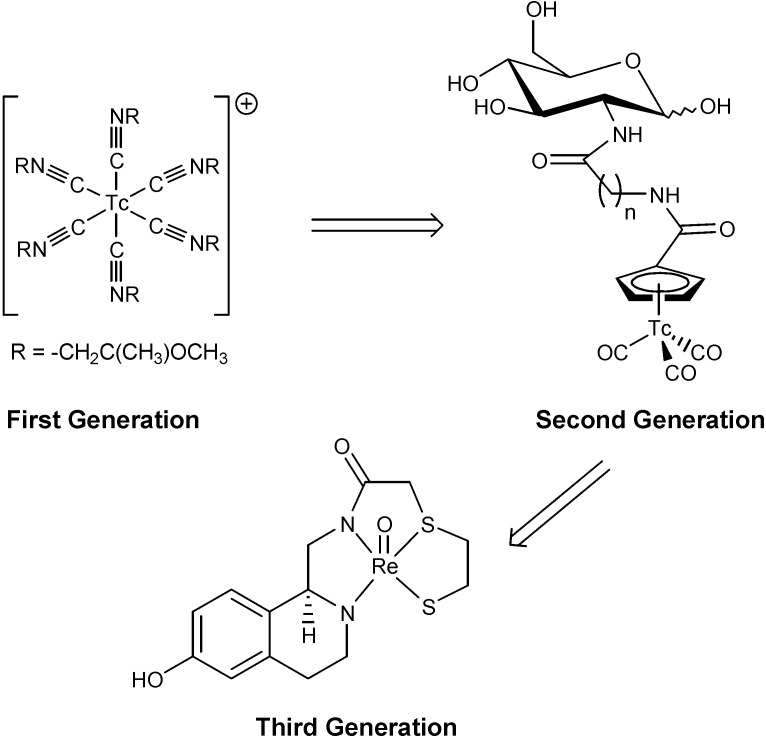
Evolution of technetium based radiopharmaceuticals.

Radiopharmaceuticals have evolved from simple metal complexes (first generation) with simple ligands, to higher complexity ligands or even ligands derived from biomolecules, mimicking lipophilic and structural properties to increase biocompatibility, biodistribution and tissue recognition specificity. The last two generations have reached clinical application, although chemically are harder targets to achieve (Figure 29) [80].

### B.2. Technetium Complexes with O-Containing Ligands

The chemistry of Tc octahedral oxo complexes has been recently reviewed [86]. The synthesis and characterization of neutral complexes *fac*-[Tc(CO)_3_(PR_3_)((L)] and *cis*-[Tc(CO)_3_(PR_3_)(L)], with PR_3_ = triphenylphosphine or methyldiphenylphosphine and L = acetylacetone or curcumin as the OO donor ligand, has been reported [87]. Preparation was achieved from the corresponding intermediate aqua complexes [Tc(CO)_3_(H_2_O)(L)] in the presence of the appropriate tertiary phosphine, which replaced the labile water molecule at room temperature. Under reflux conditions, a second phosphine ligand displace a carbonyl, generating the bisphosphine complex *cis-trans-*[Tc(CO)_2_(PR_3_)_2_(L)] in almost quantitative yield. Selective binding to beta-amyloid plaques for both the monophosphine and the bisphosphine complexes of curcumin was reported, making these compounds potentially useful for pharmacological uses.

**Figure 30:**
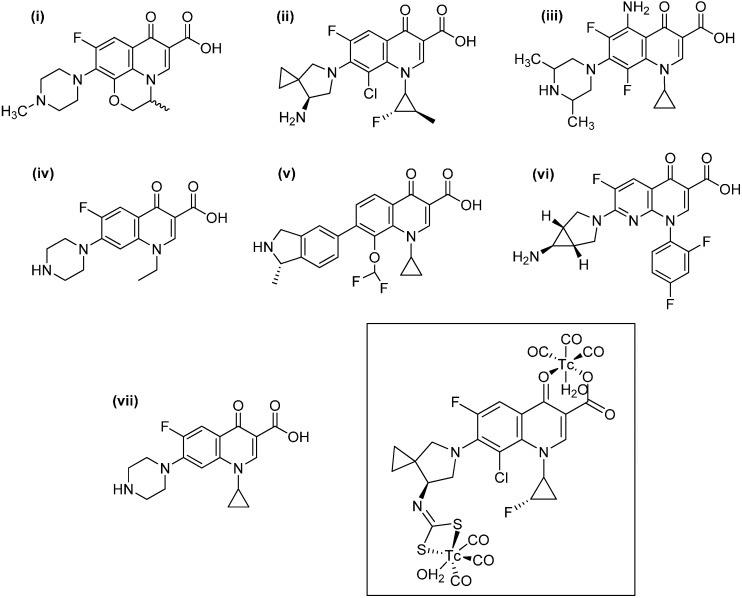
Antibiotics containing the fluoroquinolone skeleton used to prepare potential infection radiotracer imaging agents: (i) Oxoflacin; (ii) Sitafloxacin; (iii) Sparafloxacin; (iv) Norfloxacin; (v) Garenoxacin; (vi) Trovafloxacin; (vii) Ciprofloxacin. In the box, chelating modes of fluoroquinolones and its dicarbamathe derivatives are shown for sitafloxacin.

**Figure 31:**
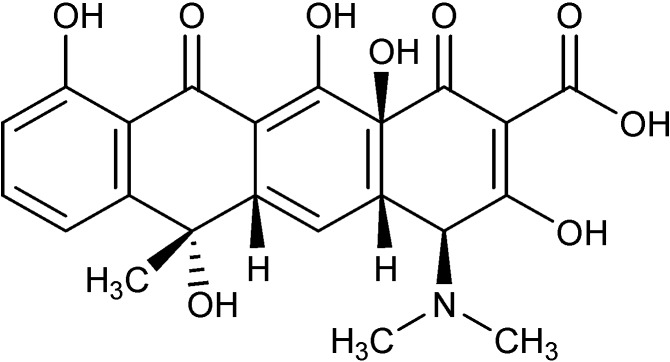
Molecular structure of doxycycline.

Antibiotics such as ofloxacin, sitafloxacin, sparafloxacin, norfloxacin, garenoxacin, trovafloxacin, ciprofloxacin and norfloxacin, have been explored as ligands to prepare technetium-99m tricarbonyl complexes (Figure 30). These complexes have been tested against *S. aureus* as a bacterial infection model both *in vitro* (bacterial cultures) and *in vivo* (infected rats). All these molecules share a common fluoroquinolone skeleton. Fluoroquinolones are broad-spectrum antibiotics with good oral absorption and excellent bioavailability. They possess a carboxylic acid function at the 3-position and a carbonyl oxygen atom at the 4-position, becoming potentially good bidentate chelating ligands toward metal ions. Preparation of the dithiocarbamate derivative in some cases was explored, in order to use the-CS_2_ fragment as a coordinating moiety toward the ^99m^Tc ion. Good biodistribution and high accumulation in the infected region make these complexes suitable for applications as radiotracer for infection imaging [88,89,90,91,92,93,94,95,96]. Doxycycline, another antibiotic used for the treatment of several infections and part of the tetracycline class, has been also labeled with ^99m^Tc to explore its use as a radiotracer for infection imaging [97]. It also has several chelating moieties, which makes it a versatile ligand for coordination to metal ions (Figure 31). Stability, sterility and *in vivo* distribution in an animal model (rats) were performed, finding high uptake in bacterial infection site, yielding promising results.

### B.3. Technetium Complexes with N-, O-, P- or S-Containing Ligands

**Figure 32:**
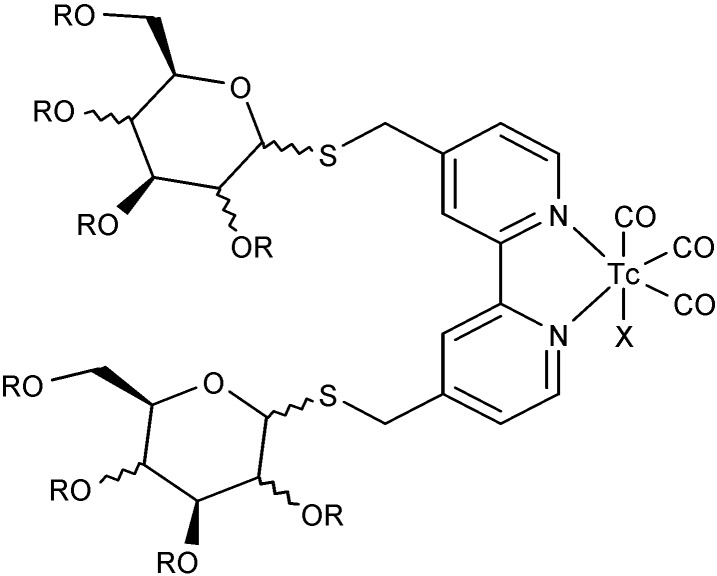
Example of bidentate ^99m^Tc complex: a sugar substituted bipyridine Tc-complex.

Several technetium coordination complexes with chelating ligands (bi-, tri- and tetradentate) containing at least a nitrogen atom have been reported in the past decade. These ligands seek to overcome problems related to the use of the Tc(I) core as a radiotracer in a metal complex, as they need to be hydrophilic. However, most of the chelating agents are lipophilic, contributing to a poor pharmacokinetic performance. Several of those chelating ligands, coordinate to different ^99m^Tc targets, such as [Tc(CO)_3_]^−^ or [TcO_3_]^+^. For example, the reaction between [TcO_4_]^−^ and the strong Lewis acids benzoyl chloride and BF_3_**^.^**OEt_2_, was explored for the synthesis of complexes containing the [TcO_3_]^−^core with ligands such as 2,2'-bipyridine, 1,10-phenantroline, di-1*H*-pyrazol-1-yl acetate, *bis*(3,5-dimethyl-1*H*-pyrazol-1-yl)acetate, 1,1,1-methanetriyltris(3,5-dimethyl-1*H*-pyrazole), and their ^99^Tc NMR spectra recorded; their rhenium analogues were also structurally characterized [98] . Water soluble ^99m^Tc complexes with sugar-substituted bipyridine complexes obtained from the reaction of 4,4'-dibromomethyl-2,2'-bipyridine with 2,3,4,6-tetra-O-acetyl-β-D-glucopyranosylthiol, 2,3,4,6-tetra-O-acetyl-β-D-galactopyranosylthiol or 2,3,4,6-tetra-O-acetyl-α-D-thioacetylmannopyranoside were obtained and fully characterized (Figure 32).

The complexes are stable for several hours in the presence of coordinating ligands (histidine), showing partial ligand-exchange after 24 h [99]. In other work, the preparation of ^99m^Tc complex with a bidentate 6-(pyridine-2-methylimine)-4-[3-bromophenyl)amino]-quinazoline ligands was reported recently [100]. This metal complex was explored for use as a biomarker for EGFR positive tumors, observing inhibition of the EGFR autophosphorylation. Finally, a series of myocardial perfusion imaging agents was prepared using different mono- and bidentate ligands (imidazole, 1,10-phenanthroline, 2,2-bipyridine) labeled with tricarbonyl-^99m^Tc were reported and fully characterized [101]. Dihydropyrimidinone [102] and bemzamidoxime [103] derivatives were synthesized and their ^99m^Tc complexes prepared using stannous chloride as reducing agent. Their potential uses are as radiotracers for infections (*E. coli*) or lung radioimaging, showing good biodistribution and stability. The complexes were prepared from sodium pertechnate or ^99m^Tc-glucoheptonate, with high radiochemical yields.

**Figure 33:**
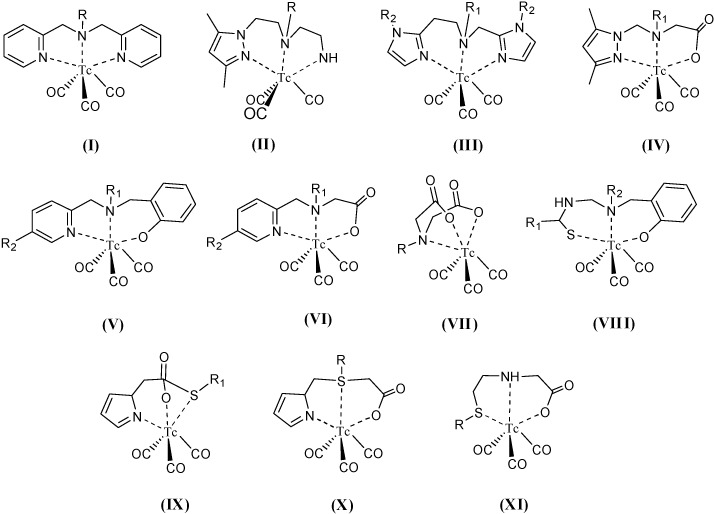
Type of chelating group present at the ^99m^Tc complexes shown in Table 1.

**Figure 34:**
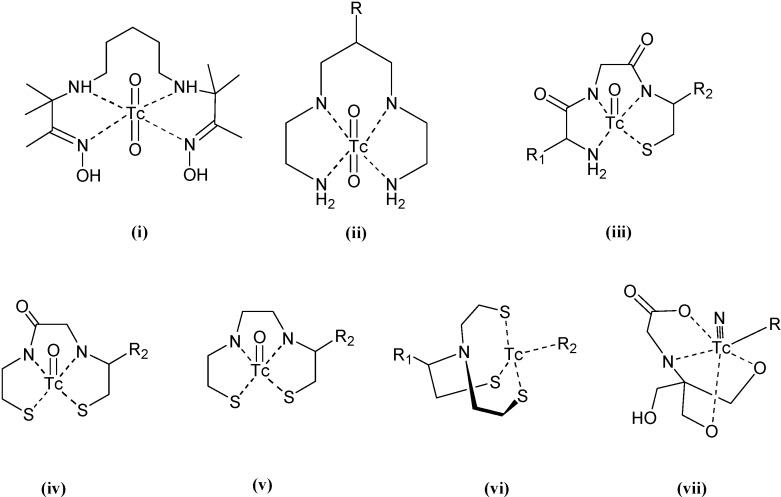
Type of chelating group present at the ^99m^Tc complexes shown in Table 2.

Other ^99m^Tc complexes with tridentate and tetradentate ligands containing at least a nitrogen donor atom, were prepared, characterized and their use as biomedical radiotracer agents explored. Figure 33 and Figure 34 show the common chelating groups used to tri-coordinate and tetra-coordinate technetium into a complex, respectively. Table 1 and Table 2 summarize some selected examples of this type of ^99m^Tc chelating ligands reported in the lapse time of this review.

**Table 1:** Synthesis of ^99m^Tc complexes with chelating tridentate N containing ligands.

Denticity and Donor Atoms	Conjugated Ligand	Chelating Group	Potential Medical Use	Ref.
N,N,N	Benzothiazole and stilbene	II	Brain imaging	[104]
	Lys-NHCONH-Glu inhibitor	II	Imaging of prostate specific membrane antigen (PSMA)	[105]
	Cyclo-[Arg-Gly-Asp-D-Tyr-Lys(PZ)]	I	Integrin receptors in tumor cells and neovasculature	[106]
	Ala-NLe-cyclo[Asp-His-DPhe-Arg-Trp-Lys]-NH_2_	I	Imaging of melanocortin type 1 receptor (MC1R) in melanoma tumors	[107]
	Lysine aminoacid derivatives conjugated to octreotide	III	Tumor imaging	[108]
	16-mer peptide nucleic acid sequence H-A GAT CAT GCC CGG CAT-Lys-NH_2_	I	Radioimaging human neuroblastoma cells	[109]
	Diethyl phosphonate, phosphoric acid and bisphosphonic acid derivatives	I	Bone imaging	[110]
	l-Arg conjugates	I	Monitoring of *in vivo* activity of inducible nitric oxide synthase (iNOS)	[111]
	Lisinopril	II	Imaging of angiotensin-convering enzyme (ACE) for hearth failure monitoring	[112]
	Insulin	II	Tracing insulin biochemistry *in vivo*	[113]
	Aliphatic or aromatic ethers	I, II	Cardiac imaging	[114]
	Glu-urea-Lys, Glu-urea-Glu	I, II	Imaging of prostate specific membrane antigen (PSMA)	[115]
	DNA intercalator and bomesin analogue	II	Imaging gastrin releasing peptide receptor (GRPr) and Auger therapy	[116]
	Duanidino, N-hydroxyguanidine, N-methylguanidine, N-nitroguanidine or S-methylisothiurea moieties	II	iNOS visualization	[117]
	Ac-DEVD-R110-D-SAAC-Fmoc	II	Monitoring of apoptosis	[118]
	Pamidronate and alendronate	I	Bone imaging	[119]
	Bile acid	I	Radiopharmaceuticals for hepatobiliary diseases, liver tumor and intestinal cancer	[120]
N,N,O	Pyridyl-*tert*-nitrogen-phenol ligand	IV	Radiolabeling agents	[121]
	Glucosamine	IV	Radiolabeling of glucose biochemistry	[122,123]
	4-Nitrobenzyl moiety	IV	Bioreductive diagnostic radiopharmaceutical	[124]
	15-[N-(hydroxycarbonylmethyl)-2-picolylamino)pentadecanoic acid	V	Radiotracer for evaluation of fatty acid metabolism in myocardium	[125]
	Estradiol	VI	Imaging agent for estrogen receptor in tumor cells	[126]
	Triphenylphosphine	VI	Radioactive metalloprobes for *in vivo* monitoring of mitochondira	[127]
	Quinazoline derivatives	IV	Biomarker for EGFR-TK positive tumors	[128]
N,O,O	2- and 4-Nitroimidazole	VII	Imaging hypoxic cells	[129,130,131]
	Glucosamino-Asp-cyclic(Arg-Gly-Asp-D-Phe-Lys)	VII	Angiogenesis imaging agent	[132]
N,O,S	Benzoyl thiourea	VIII	Radiopharmaceuticals	[133]
	Histidine derivatives	IX	Radiopharmaceuticals	[134]
	3-(carboxymethylthio)-3-(1H-imidazol-4-yl)propanoic acid	X	Radiopharmaceuticals	[135]
	Thymidine	XI	Monitoring activity human thymidine kinase type 1	[136]

**Table 2:** Synthesis of ^99m^Tc complexes with chelating tetradentate N-containing ligands.

Denticity and Donor Atoms	Conjugated Ligand	Chelating Group	Potential Medical Use	Ref.
N,N,N,N	2,2'-(1,4-diaminobutane)b8s(2-methyl-3-butanone) dioxime	i	Hypoxia markers	[137]
	1,4,8,11-tetra-azaundecane derivatives	ii	SPECT imaging probes for tumor imaging	[138]
N,N,N,S	2-nitroimidazole derivative	iii	Tumor hypoxia	[139]
	Probestin derivative	iii	Imaging aminipepetidase N (APN) expression *in vivo*	[140]
N,N,S,S	Benzothiazole aniline, pyridyl benzofuran, phenylbenzoxazole, dibenzylideneacetone derivatives	iv, v	Beta-amyloid plaques imaging in brain	[141,142,143,144]
	2-quinolinecarboxamide	iv	Peripheral benzodiazepine receptor (PBR) imaging	[145]
N,S,S,S	Fatty acid derivatives	vi	Myocardial metabolism imaging	[146,147]
	5-nitroimidazole derivatives	vi	Hypoxia tumor imaging	[148]
N,O,O,O	Pteroyl-Lys derivative	vii	Tumor imaging	[149]

**Figure 35:**
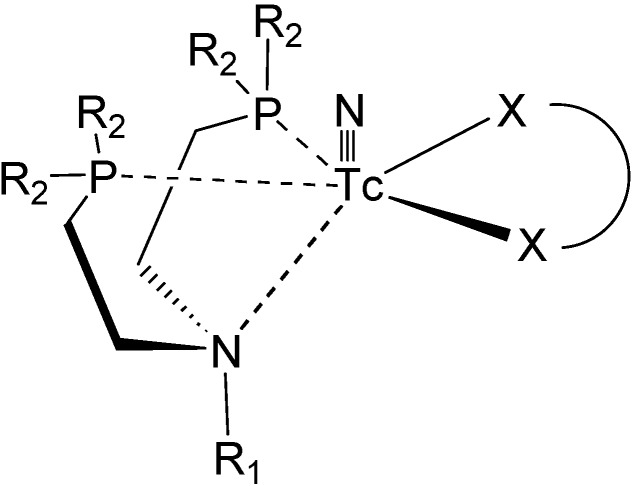
Schematic representation of the chelating core of PNP tridentate ligands.

Tridentate complexes, with PNP chelating ligand as shown in Figure 35, have been reported. A couple of works reported the preparation of a lipophilic cationic ^99m^Tc-DBODC complex (DBODC = dimethoxypropylphosphinoethyl)ethoxyethylamine) which was investigated as myocardial imaging agent [150,151,152]. The impact of bidentate chelators on lipophilicity, stability and biodistribution in Sprague-Dawley rats of a cationic ^99m^Tc-nitrido complex with a similar PNP tridentate ligand was studied; it was found that the metal complex was a very promising candidate for further preclinical studies in other animal models [153]. In a related work, a dithiocarbamate metronidazole derivative, potassium 2-(2-methyl-5-nitro-1*H*-imidazolyl)ethyldithiocarbamate, was synthesized and its complex with technetium was prepared in order to evaluate its potential as a tumor hypoxia marker [154]. The functionalization of the PNP tridentate ligand with fatty acid ligands was also explored, for being used as labelling agents to follow myocardial metabolism. The fatty acid derivatives were attached to one terminus of the carbon chain into a dithiocarbamate fragment [155].

Other metal complexes with different donor atoms were also reported. For example, carboxyl-rich thioether tridentate ligands were used to form technetium complexes and were used to measure effective renal plasma flow in rats. The complexes were formed from the reaction among [Tc(CO)_3_(H_2_O)_3_]^+^ at 70 °C and carboxymethylmercaptosuccinic acid or thiodisuccinic acid [156]. A bulky alkylphosphino-thiol bidentate ligand was used to form a complex with technetium; when reacted in the presence of a dithiocarbamate it was found that stable dissymmetrical mixed-substituted complexes were formed [157]. It was reported that these complexes may have potential applications as radiopharmaceuticals for imaging and therapy. Finally, a technetium-diethyl dithiocarbamate (DEDT) complex was prepared by a two-step procedure and studied as a potential brain radiopharmaceutical for brain imaging. Biodistribution in mice indicate that the complex is able to penetrate through the blood-brain-barrier (BBB), suggesting it may be potentially useful as a brain perfusion tracer [158].

From 2007 to the date, several research groups have explored the preparation of Tc(I) complexes based on ligands obtained by click chemistry. Click chemistry provides a useful synthetic tool for the preparation of multifunctional radiopharmaceuticals for several potential biomedical applications. With that in mind, Huisgen click chemistry and monodentate phosphine ligands have been used for biomolecule incorporation on ^99m^Tc complexes [159]. Bombesin analogues were prepared by a “click approach”, using Cu(I)-catalyzed cycloaddition to obtain a new series of triazole-based chelating systems for labeling ^99m^Tc(CO)_3_ moieties, which presented good biodistribution and improved tumor detection [160,161]. A bidentate ligand containing a bioactive pharmacophore, (2-methoxyphenyl)piperazine, has been prepared by this synthetic strategy, to further obtain a lipophilic technetium complex, potentially useful as a CNS imaging agent [162]. In other work, by the same group, the first example of a tridentate ligand obtained by click chemistry, was reported, and it was used to form a ^99m^Tc(CO)_3_ complex for radioimaging [163]. Finally, a tetradentate ligand able to form Tc(V) complexes was obtained by a “click-to-chelate” strategy, and its ability for being used as a *in vivo* radiotracer explored successfully [164].

### B.4. Technetium Organometallic Complexes

**Figure 36:**
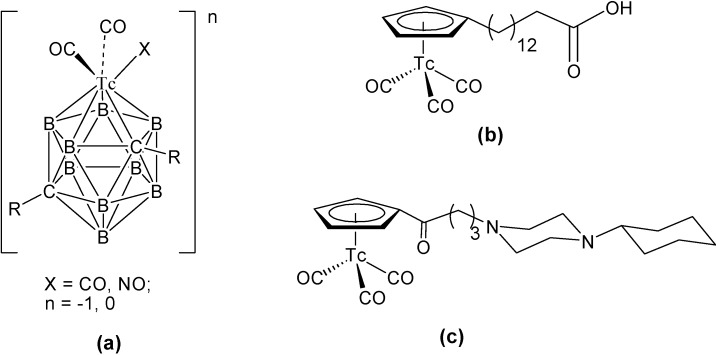
Selected technetium organometallic compounds.

Technetium organometallic compounds were prepared as potential use for imaging and cancer therapy. A ferrocenyl triarylbutane derivative labeled with ^99m^Tc by metal exchange reaction with [TcO_4_]^−^ was synthesized and its *in vivo* biodistribution was determined in female Wistar rats, with promising results [165]. Causey and coworkers reported the synthesis and evaluation of mono- and di-aryl technetium metallocarborane derivatives [(RR'C_2_B_9_H_9_)Tc(CO)_2_(NO)] (R = *p*-PhOH, R' = H) as a new class of probes for estrogen receptors [166]. The technetium-carborane was generated using a cage isomerization process, in high yield (84%). In a closely related research, a functionalized carborane complex with ^99m^Tc core, prepared by a microwave assisted approach, was studied for potential use as organometallic probes for *in vitro* and *in vivo* correlated imaging [167]. In a different work, long chain fatty acid analogs, labeled with ^99m^Tc were prepared by linking at the omega-position of pentadecanoic acid acyclopentadienyltricarbonyltechnetium fragment [168]. The novel, lipophilic complex, was injected into rats and it was found to accumulate in myocardial tissue. It is a promising radiotracer for myocardial metabolism monitoring. In 2007, Miroslavov *et al.* developed a reasonable yield synthesis to prepare [Tc(CO)_5_X] (X = Cl^−^, Br^−^). From this compound, they were able to prepare the *t*-butyisocyanide and tripheynlphosphine derivatives, by halide substitution [169]. The preparation of a new cytectreene of general formula RCpTc(CO)_3_ (R = C_6_H_5_NHCO, Cp = cyclopentadyenyl) was prepared from N-phenylferrocenecarboxamide. The ^99m^Tc complex was lipophilic enough to cross the BBB, making it an interesting base for the development of brain perfusion imaging agents [170]. Finally, in the quest for novel organometallic ^99m^Tc imaging agents, water stable N-heterocyclic carbine complexes were prepared by the reaction of [TcO(glyc)_2_]^−^ (glyc = ethyleneglycolato) with 1,3-dimethylimidazoline-2-ylidene, 1,1'-methyelen-3,3'-dimethyl-4,40-dimidazoline-2,2'-diyldene and 1,1'-methylene-3,3'-diethyl-4,4'-diimidazoline-2,20-diyldene in THF. Bidentate NHCs complexes were water-stable over a broad pH range, paves the way for the design of novel radiopharmaceuticals based on NHC complexes [171]. Figure 36 shows some selected examples of these technetium organometallic compounds.

### B.5. Applications of Technetium Labeling to Nanomaterials

In the dawn of nanoscience and nanotechnology, radiolabeled nanomaterials are becoming a common practice in the field. Multifunctional nanomaterials can simultaneously be used for diagnostic and therapy in a relatively young field called theranostics [172]. Then, radiotracers incorporated into nanomaterials make them useful as novel medical imaging agents, with the ability to penetrate through several biological barriers, fine tune their selectivity to specific targets and to modulate their biodistribution. This field is still young, but it can be prognosticated that in the future more advances and contributions will be available in the scientific literature. For example, PLA-PEG (polylactic acid-polyethylene oxide) nanocapsules labeled with ^99m^Tc-HMPAO (hexamethylpropylene-amine oxime) were prepared and their physical properties (size, size distribution, homogeneity) were determined by photon correlation spectroscopy and zeta potential by laser Doppler anemometry [173]. The results suggest that the radiolabeled nanocapsules were more stable against label leakage in the presence of proteins and could have better performances as radiotracers *in vivo*. In another work, polylactide-co-glycolide (PLGA) nanoparticles containing chloramphenicol were obtained by emulsification solvent evaporation, using polyvinylalcohol (PVA) or polysorbate-80 (PS-80) as surfactants. The nanoparticles were radiolabeled with ^99m^Tc by stannous reduction and their biodistribution after intravenous administration in mice was followed. Brain uptake was high, with low accumulation in bone marrow. The results are promising for the use of these systems for drug delivery and controlled release agents [174]. The use of technetium as radiolabeling agent to study the biodistribution of self-assembling protein nanoparticles allowed to determine their pharmacokinetic properties *in vivo*, in order to evaluate their usefulness as vaccine platforms [175]. In another work, pullulan acetate nanoparticles (PAN) were prepared by dialysis and radiolabeled with ^99m^Tc with a 98% efficiency. The hydrophobic, spherical particles, with seizes in the range from 50 to 130 nm, were stable in aqueous suspensions and may be efficient for intratumoral administration [176]. Dendrimers belong to a special class of nanostructured materials with growing interest for pharmaceutical and biomedical use. Partially acetylated generation five polyamidoamine (PAMAM) dendrimer (G5-Ac) was reacted with biotin and 2-(*p*-isothiocyanatobenzyl)-6-methyl-diethylenetriamine-pentaacetic acid and avidin to form a dendrimer-avidin conjugate, which was radiolabeled with ^99m^Tc. The nanostructured conjugate was evaluated for *in vitro* cellular uptake and biodistribution [177]. Finally, the preparation, characterization and biodistribution of letrozole loaded PLGA nanoparticles in tumor bearing mice was recently reported [178]. The PLGA nanoparticles were prepared by the solvent evaporation technique and characterized by TEM and DLS; radiolabeling with technetium was achieved with high efficiency and biodistribution indicate that the letrazole loaded nanoparticles present higher tumor uptake than usual drug delivery vehicles.

## Conclusions

During the last 7–10 years (2007–2014), nearly 2,500 research papers have been published containing theoretical and experimental results on the chemistry of actinide and technetium metallic elements. They have revealed novel and interesting physical and chemical properties of their coordination and organometallic chemistry, in particular revealing fundamental information on their unusual molecular and electronic structures and reactivity. For instance, the highest observed Werner coordination number (15) has been found in a Th complex with formula [Th(H_3_BNMe_2_BH_3_)_4_]. Most of these complexes are formed by chelating, polydentate ligands containg O- and N-donor centers, but several heteroatom mixed ligands containing S-, P- ligands have been also explored. The solid state structures of these compounds has been extensively studied by single crystal X-ray crystallography in order to determine the variable and unique coordination modes of the several functional groups included into the ligands in order to coordinate toward the radioactive metal atoms. Talking about their usefulness, the design of complex, chelating, multidentate ligands can be applied in the processes of nuclear waste remediation (*i.e.*, recycling of nuclear fuel and the selective separation of actinides and other fission products from waste solutions). Applications in analytical chemistry as specific ligands forrecongnition and determination of actinides in solutions has been also reported. Their rich and unique organometallic chemistry has also been heavily explored, and without any doubt still will keep showing in the future novel compounds with extraordinary properties and structures. Their bioactive properties, resulting from the radioactive and spontaneous emission of alpha or beta particles and/or gamma radiation, have been also explored for the design of novel antimicrobial and anti-fungalcompounds. In particular, the chemistry of technetium-99m (^99m^Tc) short-lived metastable nuclide has been exploited for the preparation of metal complexeswith lipophilic ligands for brain and heart radioimaging, asa well as for radiolabeling antibiotics, steroids, peptides and other bioactive molecules, not only for tracking their fate into the organisms, which is of great help for pharmaco-kinetic studies or the understanding of metabolic pathways, but also for the preparation of novel *in vivo* imaging agents for diagnostics and therapy. A very promising field for the application of ^99m^Tc or Ac complexes or radiotracers is in the very explosive field of nanoscience and nanotechnology, in particular to the use of nanomaterials in health (nanomedicine). Their application for monitoring the biodistribution, accumulation and metabolism of radiolabeled nanomaterials designed for drug transport and controlled releasing, theranostic agents (simultaneous diagnostics and therapy agents in one material), medical imaging and other related biomedical applications, without any doubt will result in many interesting contributions in the near and far future, which will enrich the already extraordinary broad and productive field of research of radioactive metal complexes.
